# Comprehensive Analysis of m6A‐Related Programmed Cell Death Genes Unveils a Novel Prognostic Model for Lung Adenocarcinoma

**DOI:** 10.1111/jcmm.70255

**Published:** 2025-01-19

**Authors:** Xiao Zhang, Yaolin Cao, Jiatao Liu, Wei Wang, Qiuyue Yan, Zhibo Wang

**Affiliations:** ^1^ Department of Thoracic Surgery The First Affiliated Hospital of Nanjing Medical University Nanjing China; ^2^ Department of Respiratory Diseases The Affiliated Huai'an Hospital of Xuzhou Medical University Huai'an Jiangsu China

**Keywords:** lung adenocarcinoma, machine learning, N6‐methyladenosine, precision oncology, programmed cell death

## Abstract

Lung adenocarcinoma (LUAD) involves complex dysregulated cellular processes, including programmed cell death (PCD), influenced by N6‐methyladenosine (m6A) RNA modification. This study integrates bulk RNA and single‐cell sequencing data to identify 43 prognostically valuable m6A‐related PCD genes, forming the basis of a 13‐gene risk model (m6A‐related PCD signature [mPCDS]) developed using machine‐learning algorithms, including CoxBoost and SuperPC. The mPCDS demonstrated significant predictive performance across multiple validation datasets. In addition to its prognostic accuracy, mPCDS revealed distinct genomic profiles, pathway activations, associations with the tumour microenvironment and potential for predicting drug sensitivity. Experimental validation identified RCN1 as a potential oncogene driving LUAD progression and a promising therapeutic target. The mPCDS offers a new approach for LUAD risk stratification and personalised treatment strategies.

## 1 Introduction

Lung cancer is a malignant tumour that originates from the bronchial mucosa or pulmonary glandular tissue and is generally divided into non–small‐cell lung cancer (NSCLC) and small‐cell lung cancer. NSCLC includes various subtypes, such as lung adenocarcinoma (LUAD), lung squamous cell carcinoma, large cell carcinoma and adenosquamous carcinoma, with LUAD being the most frequent, making up around 40% of all diagnosed cases [1]. The causes of lung cancer are multifaceted, with somatic mutations in genes that drive tumours playing a critical role in tumour development [2, 3]. Advances in targeted gene therapies and immunotherapies, particularly with immune checkpoint inhibitors (ICI) like PD‐1/PD‐L1 inhibitors, have shown promise in improving patient survival and quality of life [4, 5]. Nonetheless, the prognosis for lung cancer remains poor, with a 5‐year survival rate of about 15%, and survival in metastatic cases is even lower, roughly 6% [6, 7, 8]. Despite the fact that gene expression profiling has been suggested to enhance survival prediction, the absence of effective prognostic biomarkers continues to pose a significant obstacle [9, 10, 11, 12, 13]. Therefore, the identification of new prognostic biomarkers is urgently needed to improve risk stratification, prediction of outcomes and personalised treatment, with the ultimate aim of increasing survival in LUAD patients.

LUAD development is influenced by both genetic and environmental factors, though recent research has emphasised the role of abnormal RNA modifications, especially N6‐methyladenosine (m6A), in cancer progression [14, 15]. The m6A modification, which involves methylation at the N6 position of adenosine, is recognised as the most common mRNA modification in eukaryotic organisms. This modification is dynamic and reversible, governed by the coordinated activity of methyltransferases (also known as ‘writers’), signal transduction proteins (‘readers’) and demethylases (‘erasers’). The m6A modification has been closely tied to RNA processing events such as splicing, export and stability, and it is regulated through co‐transcriptional mechanisms by various transcription factors [16]. Evidence has increasingly shown that m6A is essential for normal physiological functions and plays a vital role in pathological conditions. In cancer, disruptions in m6A regulation can result in significant changes to the tumour microenvironment (TME) [17, 18]. In LUAD, abnormal m6A modifications have been linked to tumour growth, metastasis and unfavourable prognosis [19, 20, 21]. m6A modifications play a critical role in lung cancer progression through multiple mechanisms. The m6A demethylase ALKBH5 suppresses tumour growth by modulating the expression and activity of oncogenic pathways like YAP, while simultaneously promoting NSCLC development by influencing the TME via the JAK2/p‐STAT3 pathway. Furthermore, m6A reader proteins like IGF2BP3 enhance cancer cell proliferation, migration and invasion by stabilising oncogenic mRNAs such as TMBIM6, highlighting the intricate role of m6A modifications in lung cancer progression and metastasis [22, 23].

Programmed cell death (PCD), which is also referred to as regulated cell death, includes several types such as apoptosis, necroptosis, ferroptosis, pyroptosis, cuproptosis, parthanatos, alkaliptosis, oxytosis, entosis, lysosome‐dependent cell death and autophagy‐dependent cell death [24, 25, 26]. The regulation of tumour growth and progression is heavily dependent on PCD. Cancer cells are able to evade or resist PCD, which is a primary factor in their unchecked growth and malignancy. When PCD pathways are disrupted, damaged or abnormal cells accumulate, contributing to tumorigenesis [27]. Recent studies have suggested that the regulation of gene expression related to PCD could be influenced by m6A modification, thereby impacting cancer development [15, 28, 29]. The interplay between m6A regulation and PCD has been explored in tumours such as colorectal and bladder cancer. The discovery of biomarkers based on m6A modifications and PCD provides new grounds for stratification and personalised treatment of cancer patients [30, 31]. However, the precise function of m6A RNA modifications in controlling PCD during the progression of LUAD remains largely unclear.

This study represents a novel exploration of the intersection between m6A modifications and PCD in LUAD, a relationship that has been largely unexplored in this context. While previous research has focused on the role of m6A in cancer progression and PCD in other tumour types, the precise function of m6A‐modified PCD genes in LUAD remains unclear. Here, we utilised both large‐scale RNA sequencing and single‐cell sequencing data to comprehensively investigate this link, applying 101 distinct combinations of machine‐learning algorithms to develop an innovative 13‐gene m6A‐related PCD signature (mPCDS). The mPCDS exhibited superior prognostic accuracy compared to existing clinical and molecular markers, underscoring its potential as a robust biomarker for LUAD. Furthermore, the strong correlation identified between mPCDS and the TME offers new insights into the selection of personalised chemotherapeutic agents, particularly in targeting the oncogene RCN1, which we identified as a critical driver of LUAD progression. These findings not only expand our understanding of the m6A‐PCD axis in LUAD but also introduce new opportunities for personalised risk stratification and treatment strategies, marking a significant advancement in the management of LUAD.

## 2 Methods

### 2.1 Data Acquisition and Preparation

The RNA sequencing data, methylation patterns, copy number variations, mutation information and clinical details of LUAD patients were sourced from The Cancer Genome Atlas (TCGA) database (https://portal.gdc.cancer.gov). The single‐cell RNA sequencing dataset GSE171145, which includes nine LUAD samples, was retrieved from the Gene Expression Omnibus (GEO) (http://www.ncbi.nlm.nih.gov/geo). To validate the model, six additional datasets were gathered from GEO: GSE11969 (*n* = 94), GSE13213 (*n* = 117), GSE2693924 (*n* = 114), GSE29016 (*n* = 39), GSE31210 (*n* = 226) and GSE72094 (*n* = 398). Data from GSE91061, which contains information from 105 melanoma patients treated with anti‐CTLA4 and anti‐PD1 therapies, as well as LUAD cases from the OAK trial, a randomised controlled trial evaluating immune response to neoadjuvant immunotherapy and chemotherapy, were also incorporated [32]. To ensure the datasets were comparable, gene expression data were standardised to transcripts per million [33]. The ComBat function from the sva package in R was used to address potential batch effects [34, 35, 36]. ComBat enhances statistical power by integrating genomic data across batches. Unlike traditional methods that assume a Gaussian distribution, ComBat employs a negative binomial regression model, which is more suitable for the skewed, over‐dispersed counts in RNA‐seq data. This approach preserves the integer nature of the data and effectively removes batch effects, allowing researchers to recover meaningful biological signals. Furthermore, at the start of the analysis, all datasets from TCGA and GEO underwent log transformation to achieve consistent data formatting.

### 2.2 Identification of Prognostic m6A‐Related PCD Genes

A thorough investigation of the existing mRNA m6A modification literature was conducted, and 23 recognised m6A regulatory genes were collected and examined to investigate different m6A methylation patterns. This group included FTO and ALKBH5 (two ‘erasers’), METTL3, METTL14, ZC3H13, RBM15B, CBLL1, WTAP, RBM15 and VIRMA (eight ‘writers’), as well as YTHDC1, YTHDC2, ELAVL1, YTHDF1, YTHDF2, YTHDF3, LRPPRC, FMR1, HNRNPC, IGF2BP1, IGF2BP2, IGF2BP3 and HNRNPA2B1 (13 ‘readers’) [37, 38, 39]. Additionally, 18 types of PCD and their primary regulatory genes were identified [40, 41]. This investigation covered genes related to various forms of PCD, including apoptosis, autophagy, anoikis, ferroptosis, necroptosis and other related pathways, leading to the establishment of a complete set of PCD‐related genes (Table S1). After eliminating 416 redundant entries, a final list of 1548 unique PCD‐related genes was generated for further study. The m6A‐related PCD genes were preliminarily identified using RNA‐seq data through Spearman correlation analysis, applying selection criteria of |*R*| > 0.3 and *p* < 0.05. Genes differentially expressed between normal and cancer tissues that were associated with m6A‐related PCD were further filtered using thresholds of Log |fold change| > 0.5 and *p* < 0.05. Finally, genes linked to prognosis were identified using Cox regression analysis and Kaplan–Meier survival analysis.

### 2.3 Single‐Cell Sequencing Data Analysis

The original expression matrix was processed using the ‘Seurat’ R package (version 4.1.3) [42], with genes required to be expressed in at least 10 cells per sample for inclusion. Low‐quality cells were removed based on specific criteria: cells expressing fewer than 200 genes, more than 6000 genes or with over 10% unique molecular identifiers (UMIs) from mitochondrial genomes were excluded [43]. The transcriptomic expression matrix of the remaining high‐quality cells was then integrated using the ‘harmony’ R package [44]. Principal component analysis (PCA) was applied to the selected highly variable genes, and the first 30 significant principal components were utilised for t‐SNE to reduce dimensionality and visualise gene expression. The ‘FindAllMarkers’ function was employed to identify differentially expressed genes within each cell subgroup, and cell types and subtypes were annotated according to the expression patterns of recognised marker genes. Various scoring methods were employed to compute the mPCD score for each cell, using the average score to represent mPCD activity. These methods include AUCell, which calculates gene set activity by determining the area under the curve (AUC) for gene expression, providing a robust metric of activity in individual cells. UCell normalises gene expression scores to capture overall activity across varying conditions. SingScore measures gene set activity in single‐cell RNA‐seq data, considering both positive and negative contributions for a nuanced assessment. ssGSEA computes enrichment scores by ranking genes to reflect their over‐representation in individual samples [45]. Finally, AddModuleScore averages the expression levels of genes in a set, normalising against control genes to provide relative activity insights. We chose these methods for their complementary strengths, allowing for a comprehensive evaluation of mPCD activity.

### 2.4 Analysis of Cell–Cell Interactions

CellChat was used to combine gene expression data and evaluate differences in the predicted intercellular communication networks [46]. Through the use of the default CellChatDB, which follows standard CellChat protocols for its ligand‐receptor database, interactions specific to different cell types were identified. Overexpressed ligands and receptors in particular cell clusters were detected, allowing for the identification of enhanced ligand‐receptor interactions related to overexpression.

### 2.5 Construction of Prognostic Model Through Machine Learning

Cells were separated into groups with high and low mPCD activity, determined by the median mPCD score, and differential gene expression between the two groups was identified using the ‘findMarker’ function. Spearman correlation analysis was performed to identify the top 150 genes most strongly associated with mPCD activity. These 150 genes were combined with significantly differentially expressed genes to create a comprehensive genomic set for further model development. In the TCGA cohort, a two‐step approach was taken to identify genes with prognostic relevance. Initial screening was carried out using the ‘survival’ R package, followed by univariate Cox regression analysis to further evaluate their prognostic significance. The findings were then used to build a predictive model via machine learning. The TCGA cohort was utilised as the training set, while GSE11969, GSE13213, GSE2693924, GSE29016, GSE31210 and GSE72094 served as validation sets. In total, 101 machine‐learning algorithm combinations were tested using tenfold cross‐validation [47, 48]. The algorithms tested included StepCox, Lasso, Ridge, plsRcox, Cox Boost, RSF, GBM, Enet, SuperPC and Survival‐SVM. The predictive model was developed within a leave‐one‐out cross‐validation framework. To further reduce the risk of overfitting in multivariate Cox regression models across different cohorts, we applied the ‘pre‐validation’ function. For each model, the Harrell's concordance index (*C*‐index) was calculated across all validation datasets. The model achieving the highest average *C*‐index in the validation sets was ultimately chosen as the optimal mPCD‐related signature (mPCDS).

### 2.6 Assessment of the Prognostic Significance and Clinical Utility of the Model

To assess the prognostic relevance and possible clinical utility of the developed mPCDS model, scores for each sample in both the training and validation groups were calculated. Samples were then classified into high and low mPCD groups based on their median score. PCA was performed using the ‘prcomp’ function to verify the separation of these groups. Kaplan–Meier survival analysis was used to further evaluate the prognostic significance of mPCDS.

To evaluate the predictive capacity of the developed model, we conducted a comparative analysis of the mPCD scores against established clinical prognostic indicators, including gender, age, tumour stage and mutation status. Each of these clinical variables was treated as a univariate predictor in separate analyses. The predictive performance of each factor was assessed using *C*‐index, which quantifies the accuracy of survival predictions. Additionally, the diagnostic performance of the mPCDS model was determined by calculating the AUC values for 1‐, 3‐ and 5‐year survival using the ‘timeROC’ R package. A thorough review of 106 LUAD‐related prognostic features was conducted, and scores were assigned to each sample based on previously published coefficients (Table S2). The predictive accuracy of these features across all cohorts was also assessed using the *C*‐index.

### 2.7 Analysis of the Mutation Landscape

Mutation data were obtained from the TCGA cohort and normalised by converting them into a binary 1‐0 matrix. Gene deletions and amplifications were sourced from the GISTIC 2.0 database (ftp://ftp.broadinstitute.org/pub/GISTIC2.0/). Tumour mutation burden (TMB) was calculated using the mPCDS scores derived from the TCGA cohort, and mutation frequencies in the high and low mPCDS groups were identified. The correlation between mPCDS risk scores and TMB was also evaluated. Prognostic differences between patient subgroups classified by both TMB and risk scores were carefully examined to better understand how these factors influence patient outcomes.

### 2.8 Gene Set Variation Analysis Enrichment Analysis

Signalling pathways and immune regulation patterns associated with mPCDS risk scores were explored using gene set variation analysis (GSVA) performed through the ssgsea method within the GSVA package [49]. This analysis focused on the enrichment of gene sets for important pathways from the Molecular Signatures Database. The study also investigated the relationship between mPCDS, the cancer immunity cycle and immune‐related predictive pathways relevant to immunotherapy.

### 2.9 Immunoinfiltration Landscape Analysis

To identify variations in immune cell populations, six different immune infiltration algorithms—EPIC, TIMER, CIBERSORT, CIBERSORT‐ABS, MCPCounter, QUANTISEQ and XCELL—were applied to analyse immune cell infiltration. A heatmap was created to visually display differences in immune cell populations and immune‐related gene expression across the different risk groups. In addition, specialised functions in the ‘ESTIMATE’ R package were used to calculate immune scores, stromal scores and estimate scores for LUAD patients [50]. This method aimed to provide a deeper understanding of the TME and its potential clinical relevance.

### 2.10 Immunotherapy Response Analysis and Drug Screening

To assess the efficacy of immunotherapy, the survival of patients who showed delayed responses after treatment was first analysed. The Tumour Immune Dysfunction and Exclusion (TIDE) algorithm was used to estimate the likelihood of individual patients responding to immunotherapy [51]. Additionally, the subclass mapping algorithm was applied to identify similarities between the risk groups and immunotherapy subgroups, helping to recognise patients who might respond to anti‐CTLA‐4 or anti‐PD‐1 treatments [52]. Clinical data from patients who received immunotherapy were taken from the OAK cohort and GSE91061. The CTRP2.0 and PRISM databases were used to identify potential therapeutic drugs for high‐risk patients by evaluating tumour drug sensitivity based on AUC values.

### 2.11 LUAD Patient Samples

Ethics approval was obtained from the ethics committee (no. 2023‐SR‐777), and six pairs of tissue samples, including tumour tissue (T) and adjacent non‐tumour tissue (N), were collected from LUAD patients who underwent tumour removal at the First Affiliated Hospital of Nanjing Medical University. All patients had not undergone prior treatment and were confirmed by pathology to have LUAD. Written informed consent was obtained from all participants regarding the collection of samples, the purpose of the study, and the possibility of publication. This was done in line with the ethical guidelines of the First Affiliated Hospital of Nanjing Medical University.

### 2.12 Immunohistochemistry

Paraffin‐embedded sections were incubated with primary antibodies (anti‐RCN1, 1:200, A21590, Abclonal, Wuhan, China) at 4°C for 24 h, followed by incubation with corresponding secondary antibodies (DAKO, Glostrup, Denmark) at 37°C for 1 h. Visualisation was conducted using an Olympus microscope (BX53F, Tokyo, Japan).

### 2.13 Western Blotting

Tissue lysates were prepared, and western blotting was performed using an anti‐RCN1 antibody (1:1000, A21590, Abclonal), followed by specific secondary antibodies (LICOR, NE, USA). β‐actin antibody (1:1000, GTX109639, GeneTex) was used as the loading control. Band intensities were analysed with ImageJ software (NIH, USA).

### 2.14 Cell Culture and siRNA Transfection

Human A549 and PC9 cell lines (ATCC, USA) were cultured in RPMI 1640 medium supplemented with 10% fetal bovine serum and 1% penicillin–streptomycin (Gibco, USA) at 37°C in a 5% CO_2_ atmosphere. To suppress RCN1 expression, A549 and PC9 cells were transfected with RCN1‐specific siRNA oligonucleotides (100 nM, Genewiz) using Lipofectamine 3000 (Invitrogen, USA), following the manufacturer's instructions. Cells transfected with scrambled siRNA (100 nM) served as controls. siRNA sequences are provided in Table S3.

### 2.15 Clone Formation Assay

After transfection, cells were plated at 7 × 10^2^ cells per well in 6‐well plates and cultured for 10 days. Cells were then washed with PBS, fixed in methanol, stained with crystal violet and imaged digitally using a Canon camera.

### 2.16 Wound Healing Assay

Transfected cells were grown in 6‐well plates until they reached 95% confluence. Wounds were created in the monolayer using plastic micropipette tips, and the cells were washed twice with PBS. Images of wound closure were taken at 0 and 48 h and analysed with ImageJ software.

### 2.17 Transwell Migration and Invasion Assay

Post‐transfection, cells were placed in the upper chamber of a Transwell plate (Corning, USA) and incubated for 24 h. Migration and invasion assays were performed with or without Matrigel coating, respectively. After the invasion, cells on the inserts were fixed, stained with crystal violet and counted under a light microscope. The number of migrated cells was statistically analysed.

### 2.18 Statistical Analyses

Statistical analyses were conducted using R software (version 4.1.3). For variables that followed a normal distribution, comparisons between two groups were made using an unpaired Student's *t*‐test. For variables that did not follow a normal distribution, the Wilcoxon rank‐sum test was used. Survival analysis was carried out using the ‘survival’ package in R, and differences in overall survival (OS) between subtypes were determined by Kaplan–Meier curves and log‐rank tests.

## 3 Results

### 3.1 Identification of m6A‐Related Genes Connected to PCD in LUAD Using Bulk‐RNA Sequencing Data

**FIGURE 1:**
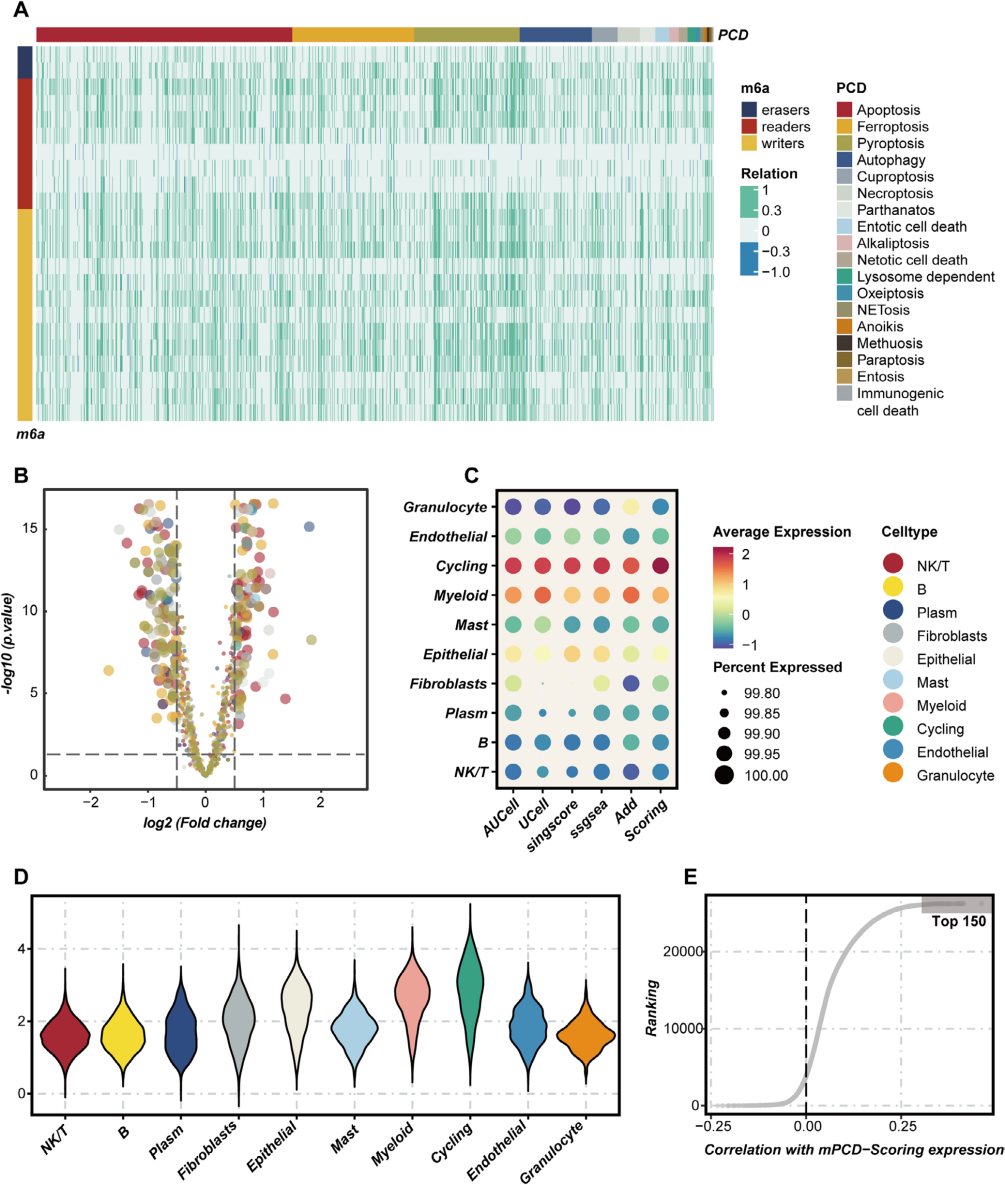
Identification of m6A‐related programmed cell death genes in lung adenocarcinoma patients. (A) Correlation analysis between m6A regulatory factors and programmed cell death (PCD) genes within the TCGA‐LUAD dataset. (B) Volcano plot depicting the differential expression of m6A‐related programmed cell death (mPCD) genes between LUAD tumour tissues and corresponding normal tissues. (C) A comprehensive assessment of m6A‐related programmed cell death gene expression across various cell types, employing five distinct scoring methodologies to minimise the biases associated with single‐algorithm approaches. (D) The violin plot illustrates the distribution of mPCD activity levels across different cell types. (E) Spearman correlation analysis was performed to identify the top 150 genes correlated with mPCD activity scores, which were subsequently utilised for further modelling and analysis.

To clarify the involvement of m6A‐related PCD in LUAD, expression profiles of 23 m6A regulatory genes in both cancerous and normal lung tissues were analysed. The findings showed that 11 regulatory genes, including three ‘writers’ and eight ‘readers’ (METTL3, RBM15, VIRMA, ELAVL1, YTHDF1, YTHDF2, LRPPRC, IGF2BP1, IGF2BP3, HNRNPC, HNRNPA2B1), were significantly upregulated in LUAD, while two ‘erasers’ and three ‘writers’ (FTO, ALKBH5, WTAP, ZC3H13, METTL14) showed downregulation (Figure S1A). The prognostic significance of these regulators was evaluated through Cox regression and Kaplan–Meier survival analyses using four survival outcomes (disease‐free interval, disease‐specific survival [DSS], OS and progression‐free interval) from the TCGA dataset. The analyses revealed that most regulators acted as risk factors, with ELAVL1 being significantly linked to all four survival outcomes (Figure S1B). In addition, the interactions between these m6A factors were examined through Spearman correlation analysis, indicating highly correlated expression patterns (Figure S1C). These results highlight the complex interplay among m6A factors. Subsequently, the expression matrix of genes related to m6A and PCD was extracted from the TCGA‐LUAD cohort, followed by a correlation analysis. Genes related to PCD that were significantly correlated with at least one m6A gene (|*R*| > 0.3 and *p* < 0.05) were classified as m6A‐related PCD genes, resulting in the identification of 1054 such genes (Figure 1A). After incorporating expression data from normal TCGA samples and applying criteria of |log_2_FC| > 0.5 and *p* < 0.05, 341 differentially expressed genes were determined (Figure 1B). Through further Cox regression and Kaplan–Meier survival analyses, 146 m6A‐related PCD genes were found to be significantly linked to OS in LUAD patients. These genes were chosen as the m6A‐related PCD gene set for further studies.

### 3.2 Identification of Key m6A‐Related Genes Involved in PCD in LUAD Patients Using Single‐Cell Sequencing Data

The study began with dimensionality reduction and clustering analyses of the LUAD single‐cell sequencing dataset GSE171145, containing nine samples. This process resulted in the identification of 14 distinct cell clusters (Figure S2A). Following that, these clusters were annotated using feature markers, which allowed the identification of various cell types, such as NK/T cells, B cells, plasma cells, fibroblasts, epithelial cells, mast cells, myeloid cells, circulating cells, granulocytes and endothelial cells (Figure S2B,C). The distribution of these cell types across different patient cohorts is displayed in Figure S2D. After annotating the cell types, the m6A‐related PCD gene set was utilised to evaluate m6A‐related PCD activity (mPCD activity) within each cell using methods like AUCell, UCell, singscore, ssgsea and AddModuleScore. The results indicated that circulating and myeloid cells exhibited increased mPCD activity, whereas B cells and NK/T cells displayed lower levels of activity (Figure 1C,D). Cells were subsequently divided into high mPCD and low mPCD groups based on median activity scores, and differentially expressed genes between these two groups were identified to further investigate the intrinsic relationships between cells with different levels of mPCD activity. Cell–cell communication analysis showed that both the number and intensity of interactions were significantly greater in the high mPCD group than in the low mPCD group (Figure S3A,B), with fibroblasts and endothelial cells showing particularly prominent communication patterns (Figure S3C). Furthermore, differences in pathway activation were also noted between the groups (Figure S3D). Finally, a Spearman correlation analysis was performed to identify the top 150 genes associated with mPCD activity scores (Figure 1E). These genes were cross‐referenced with previously identified differentially expressed genes and considered as key genes determined through single‐cell sequencing analysis.

### 3.3 Development of a Risk Signature Based on m6A‐Related PCD Genes

**FIGURE 2:**
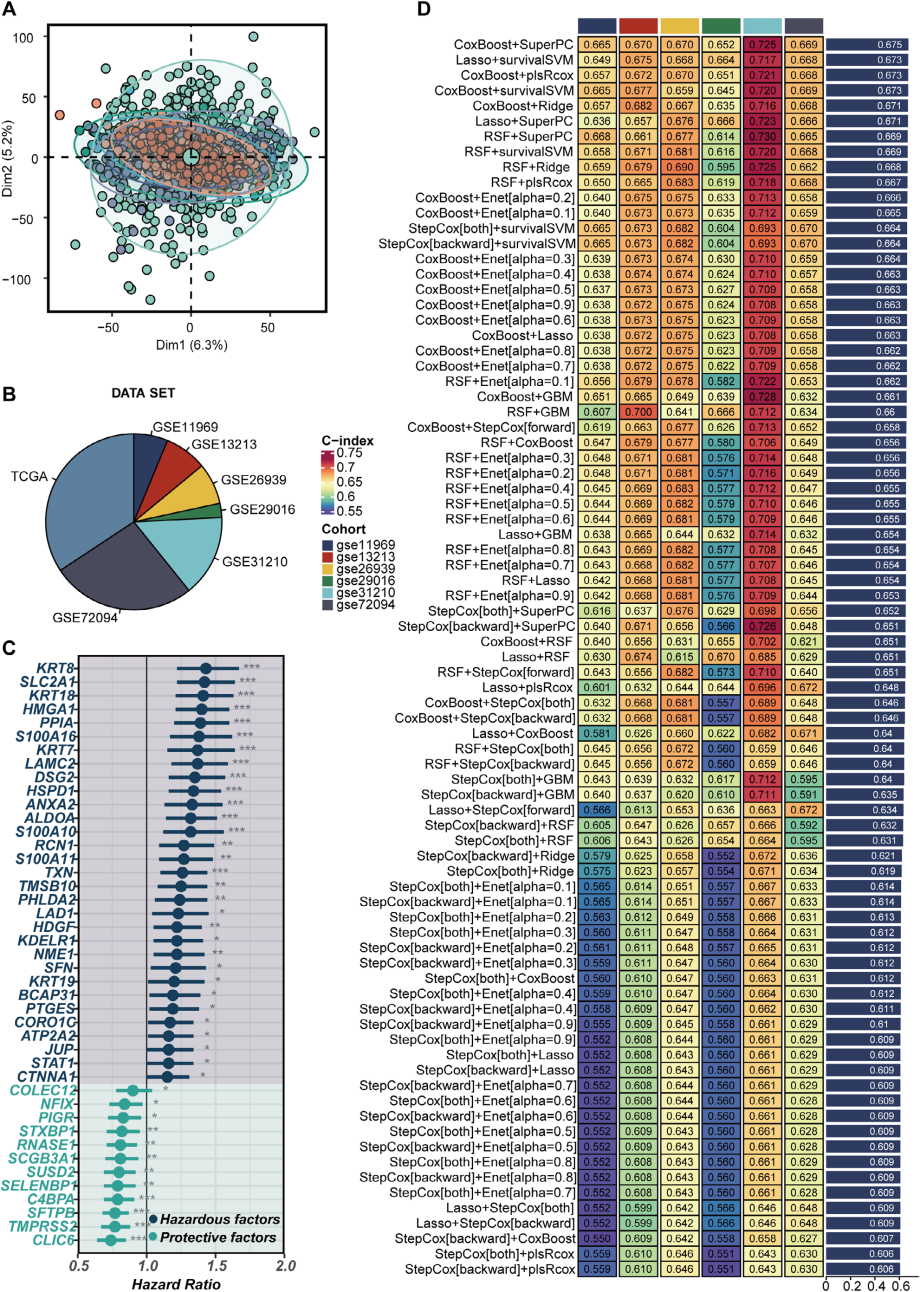
Development of the machine learning model. (A) Post‐correction distribution of samples across seven LUAD datasets, observed through the PCA algorithm. (B) A pie chart representing the seven datasets used in the construction of the model, where the TCGA dataset functions as the training set, and the other six serve as validation sets. (C) A forest plot showcasing the prognostic relevance of the 43 key genes identified through the machine‐learning algorithm analysis. (D) Optimal machine‐learning model selection was performed by calculating the average *C*‐index across six validation cohorts to determine the most effective model.

To start, batch effects from the TCGA and GEO datasets were removed (Figure 2A), and the processed data composition was shown in Figure 2B. Afterward, Kaplan–Meier survival analysis and univariate Cox regression were applied to the key genes found through single‐cell sequencing, leading to a preliminary selection. This process identified a prognostic gene set with 43 genes, including 31 risk factors and 12 protective factors (Figure 2C). To create a prognostic feature related to mPCD, a broad analysis was conducted using combinations of 10 machine‐learning algorithms. The CoxBoost + SuperPC method had the highest average *C*‐index across six validation groups, making it the preferred model for further studies (Figure 2D).

### 3.4 Prognostic Evaluation and Comparative Analysis of the mPCD‐Related Risk Signature

**FIGURE 3:**
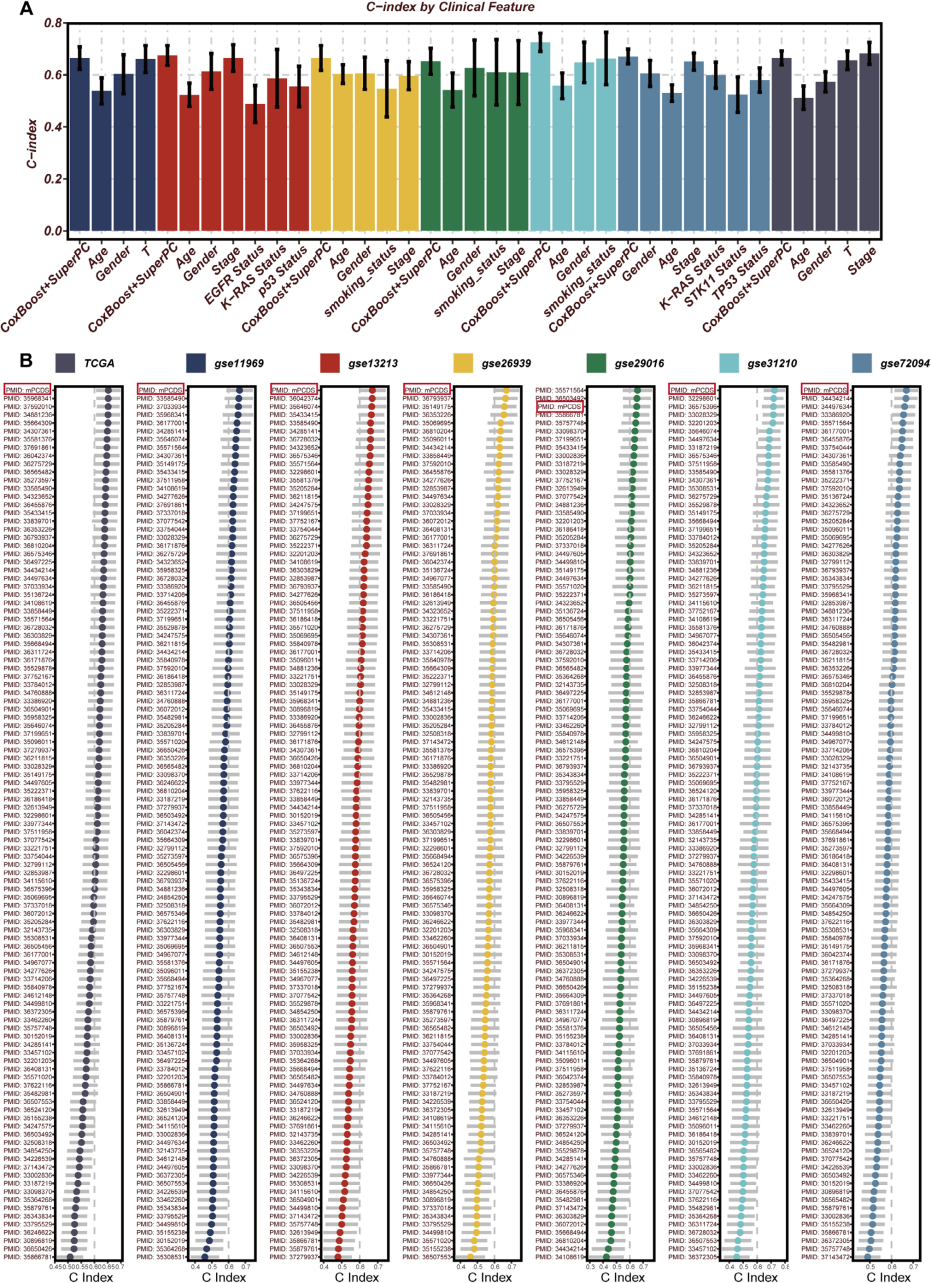
Clinical utility and prognostic value of the model. (A) Comparative analysis of the signature risk score against other clinical indicators, demonstrating its superior prognostic value. (B) Evaluation against published signatures, revealing that the signature consistently achieves the highest *C*‐index scores across multiple datasets.

To evaluate the clinical significance of mPCDS, its performance was tested across seven cohorts against several clinical factors such as age, gender, EGFR status, p53 status, stage and T stage. The mPCDS consistently showed a higher *C*‐index compared to other clinical factors (Figure 3A). By examining the coefficients of each gene in the prognostic features and comparing them with previously published LUAD prognostic features, mPCDS was found to perform consistently well across all studied cohorts, including TCGA, GSE11969, GSE13213, GSE26939, GSE29016, GSE31210 and GSE72094 (Figure 3B). The *C*‐index for mPCDS and existing LUAD prognostic signatures in predicting prognosis can be found in Table S4. The mPCDS risk score, derived from the median, was used to group patients into high‐risk and low‐risk categories. In both the TCGA training set and other validation sets, the OS of high‐risk patients was significantly lower than that of low‐risk patients (Figure S4A–G). PCA plots generated for the seven cohorts (Figure S4H–N) further showed that mPCDS effectively divided patients into two distinct groups, highlighting its ability to predict outcomes. Additionally, time‐dependent AUC analysis demonstrated that mPCDS consistently showed strong predictive ability across multiple independent groups (Figure S5). Figure S6 shows a correlation between mPCDS scores and the expression of 13 key genes in the model, indicating that aside from SFTPB and CLIC6, which had a negative correlation with mPCDS, the remaining genes were positively correlated. Further analysis of these model genes showed that patients with high expression of SLC2A1, HSPD1, HMGA1, KRT8, KRT18, ALDOA, RCN1, PPIA, TXN, KRT7 and LAMC2 had lower OS, while patients with high expression of SFTPB and CLIC6 had better outcomes, identifying SFTPB and CLIC6 as protective genes (Figure S7).

### 3.5 Genomic Mutation Patterns in Different mPCDS Score Groups

**FIGURE 4:**
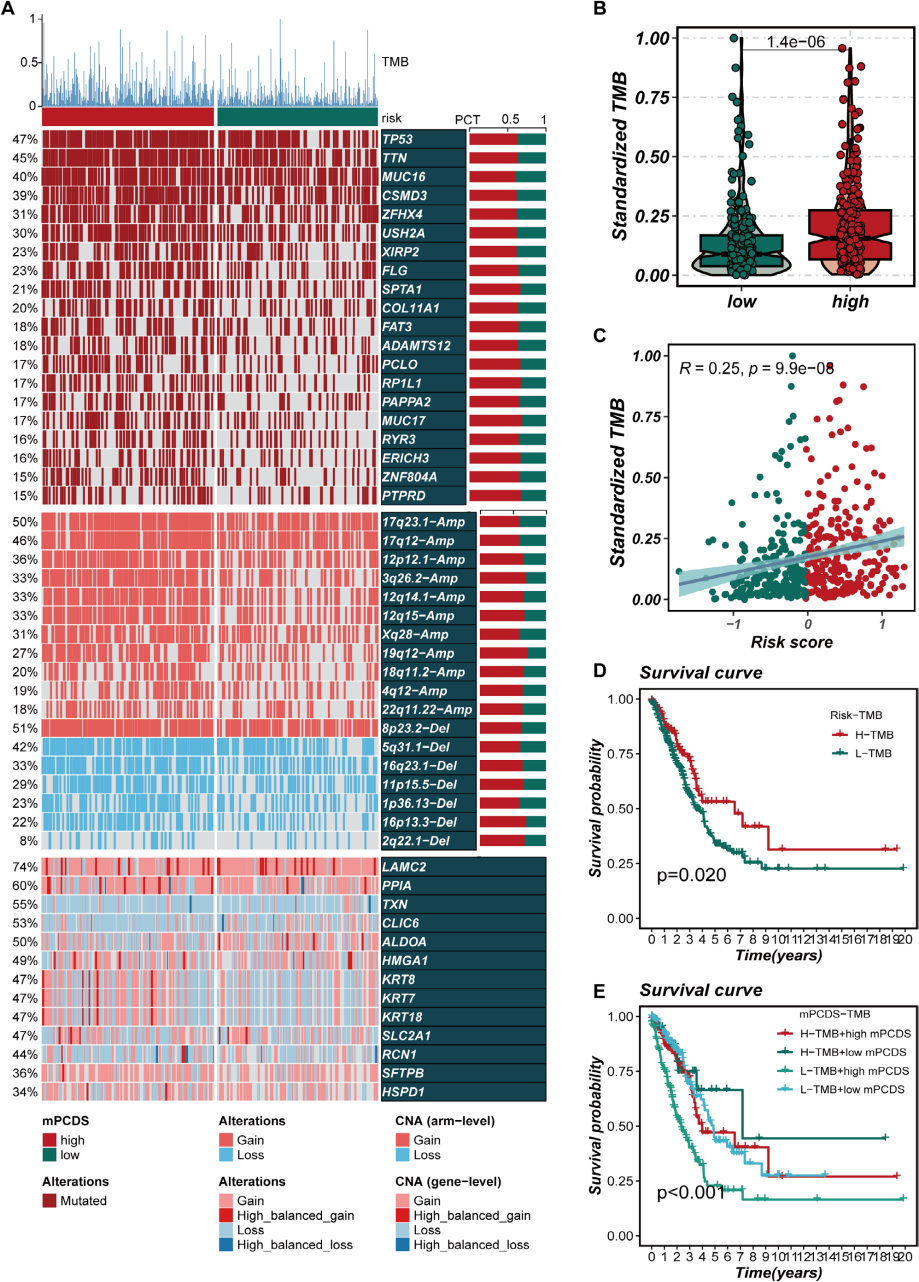
Genomic characteristics of distinct cell death–related risk score groups. (A) Overview of genomic alterations within high and low mPCDS groups. (B) Comparative analysis of normalised TMB between high and low mPCDS groups. (C) Correlation analysis between mPCDS score and TMB. (D) Survival curves depicting differences in survival outcomes between groups with high and low TMB expression. (E) Survival curves illustrating the survival differences among four distinct subgroups.

In Figure 4A, differences in TMB, mutation frequency and mutation patterns between the high‐ and low‐risk groups are shown. The genes TP53, TTN, MUC16, CSMD3 and ZFHX4 were identified as having the highest mutation rates. It was observed that the TMB in the high‐mPCDS group was significantly increased when compared to the low‐mPCDS group (Figure 4B), with a positive correlation being found between mPCDS scores and TMB (Figure 4C). Survival analysis indicated that patients in the high TMB group had better survival outcomes, whereas those in the low TMB group exhibited a notably worse prognosis (Figure 4D). Additionally, when combining TMB with the risk score, the lowest survival rates were found in the low TMB + high mPCDS group, while the best survival was noted in the high TMB + low mPCDS group (Figure 4E). These findings highlight the interaction between TMB and risk scores in affecting the prognosis of LUAD patients. The use of mPCDS as a complement to TMB allows for a more detailed stratification of patient outcomes, emphasising the importance of integrating both metrics for prognosis assessment in LUAD.

### 3.6 Biological Pathways Linked to mPCDS

**FIGURE 5:**
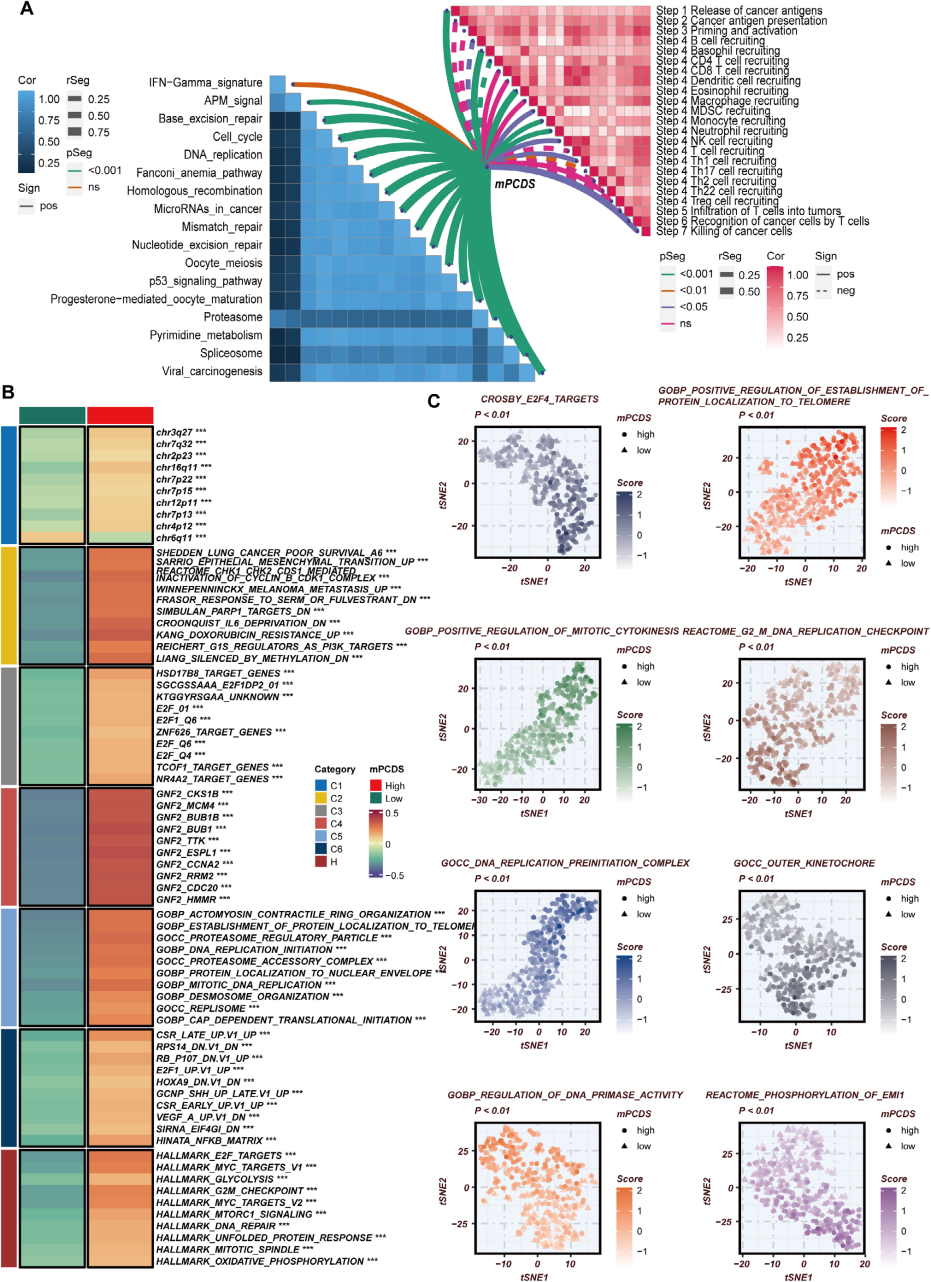
Biological pathways associated with cell death–related risk score signature. (A) Correlation analysis of mPCDS scores with tumour immune cycle pathways and oncogenic pathways, assessed via ssGSEA. (B) GSVA enrichment analysis delineating the biological characteristics distinguishing high and low mPCDS groups. (C) tSNE plot illustrating the differences in GO and KEGG pathway activities between high and low mPCDS groups. ***P < 0.001.

Due to the strong predictive ability of mPCDS for LUAD prognosis, further investigation into the potential mechanisms was conducted. GSVA was used to measure the activation levels of various biological pathways in LUAD patients. A heatmap was created to show the complex relationship between mPCDS scores, oncogenic pathways and anti‐tumour immune pathways (Figure 5A). It was found that mPCDS scores were significantly positively correlated with most oncogenic pathways. Among the seven stages of the cancer immunity cycle, significant associations between mPCDS scores and Stage 1 (cancer antigen release), Stage 4 (immune cell recruitment) and Stage 7 (cancer cell killing) were identified. Furthermore, enrichment analysis (Figure 5B) indicated that cohorts with high mPCDS scores were notably enriched in crucial pathways, including Croonquist IL6 Deprivation, Establishment of Protein Localization to Telomere, Proteasome Regulatory Particle, DNA Replication Initiation, Mitotic DNA Replication, Proteasome Accessory Complex, E2F Targets, MYC Targets and G2M Checkpoint. The pronounced enrichment of these pathways within the high mPCDS group suggests a complex network of molecular alterations that drive tumour aggressiveness in LUAD. Critical processes such as inflammation, telomere maintenance, protein degradation, DNA replication, cell cycle regulation and oncogene activation are implicated. These findings provide substantial insights into potential therapeutic targets and biomarkers for managing high‐risk LUAD patients. Further analysis, as illustrated in Figure 5C, revealed significant differences in oncogenic and immune‐related pathways between high and low mPCDS groups. Specifically, pathways such as Crosby E2F4 Targets, Positive Regulation of Establishment of Protein Localization to Telomere, Positive Regulation of Mitotic Cytokinesis, Reactome G2M DNA Replication Checkpoint, DNA Replication Preinitiation Complex, Outer Kinetochore, Regulation of DNA Primase Activity and Reactome Phosphorylation of Emi1 were significantly more enriched in the high mPCDS cohort. These results underscore the prognostic and biological significance of mPCDS in delineating the molecular and immune landscape of LUAD. The intricate interplay between oncogenic and immune pathways within the high mPCDS group highlights the complex molecular changes that contribute to tumour progression and immune evasion. The identification of these pathways lays the groundwork for developing targeted therapies and enhancing prognostic assessments for patients with high‐risk LUAD.

### 3.7 Characteristics of the Immune Microenvironment in Different mPCDS Score Groups

**FIGURE 6:**
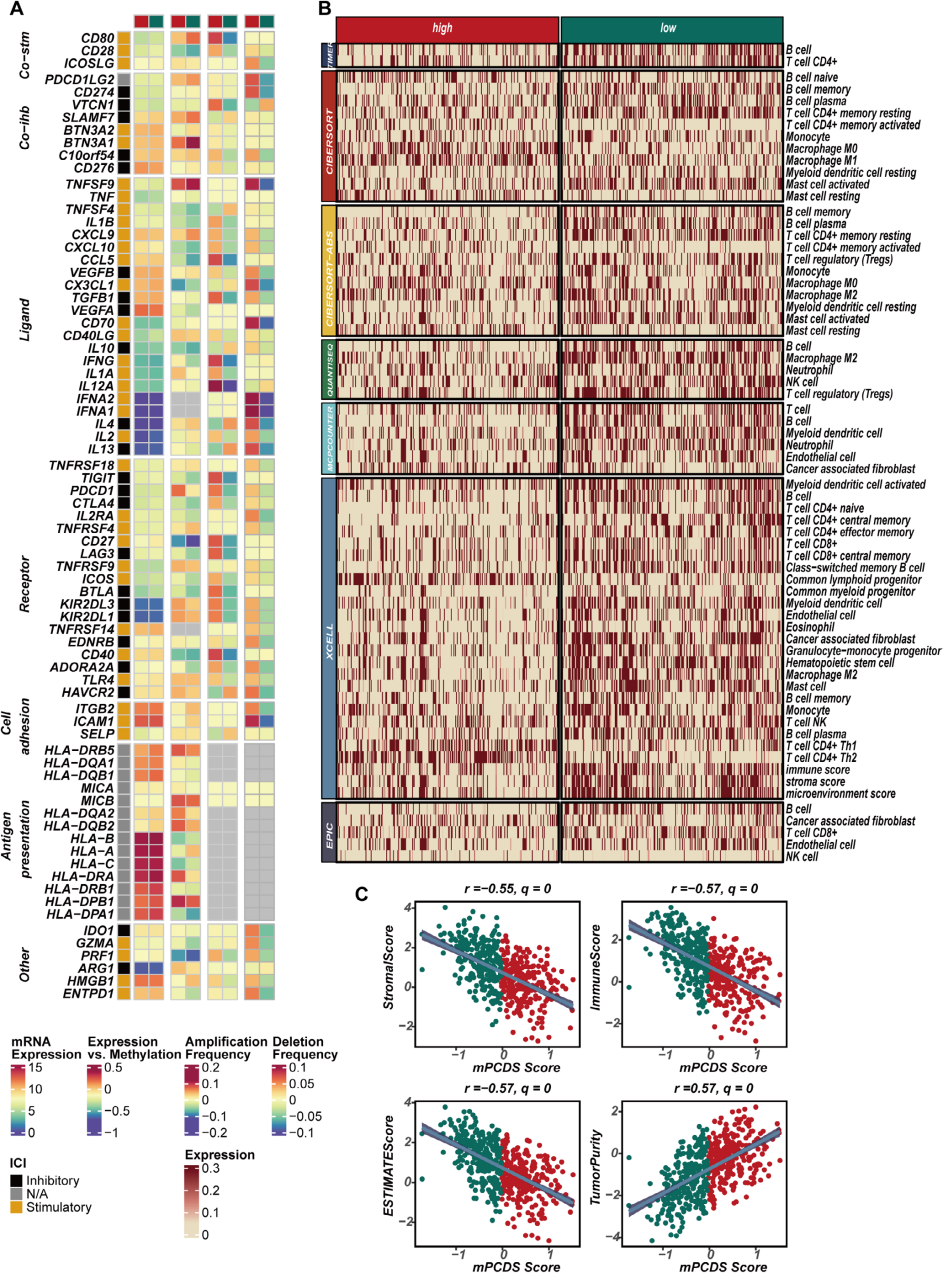
Immune microenvironment characteristics of distinct cell death–related risk score groups. (A) Differential expression analysis of immune‐related genes at the mRNA, methylation and copy number variation levels between high and low mPCDS groups. (B) Comparative assessment of immune cell infiltration between high and low mPCDS groups, using seven distinct immune infiltration algorithms. (C) Correlation analysis between mPCDS scores and immune‐related scores.

Due to the significant associations observed between mPCDS and certain inflammation‐ and immune‐related signals, a comprehensive exploration of the connection between mPCDS and the TME was carried out. Immune regulator levels in the high‐ and low‐mPCDS groups were initially evaluated (Figure 6A). It was found that, while most of the 75 immune regulatory genes exhibited similar expression across both groups, mutation rates were higher in the high‐mPCDS group, including amplifications and deletions. This suggests that, despite no significant differences in immune regulator expression, genetic instability in the high‐mPCDS group could impair the function and regulation of these genes, leading to impaired immune regulation and effectiveness, which may result in a less efficient immune response and a worse prognosis. Genes like CD274 (PD‐L1), PDCD1 (PD‐1), PDCD1LG2 (PD‐L2) and LAG3, which are central targets for ICIs, displayed increased mutation rates in the high‐mPCDS group, indicating possible differences in response to checkpoint inhibitor therapy. Changes in both mutation and expression may alter how these tumours interact with therapeutic antibodies targeting such checkpoints. CD80 and CD40, crucial for the activation of T cells, were found to be mutated in the high‐mPCDS group, which may hinder the proper activation of T cells and result in a weakened immune response. Interferon‐γ, IL12A and CCL5, which are involved in the recruitment and activation of immune cells, may have their function within the TME disrupted due to these mutations or regulatory changes. To better understand immune cell infiltration, six different methods were used to assess immune infiltration and to quantify the differences between the high‐ and low‐mPCDS groups (Figure 6B). The results consistently showed that immune cells, including B cells, CD4+ T cells and CD8+ T cells, were more prevalent in the low‐mPCDS group. This suggests that immune surveillance is more active and effective in this group. These immune cells are crucial for identifying and eliminating tumour cells, whereas in the high‐mPCDS group, the immune response is likely compromised due to genetic instability and mutations in key regulatory genes. Correlation analysis confirmed these findings, with mPCDS showing negative correlations with stromal, immune and estimated scores while being positively correlated with tumour purity (Figure 6C).

### 3.8 Role of mPCDS in Predicting Response to Immunotherapy and Drug Sensitivity

**FIGURE 7:**
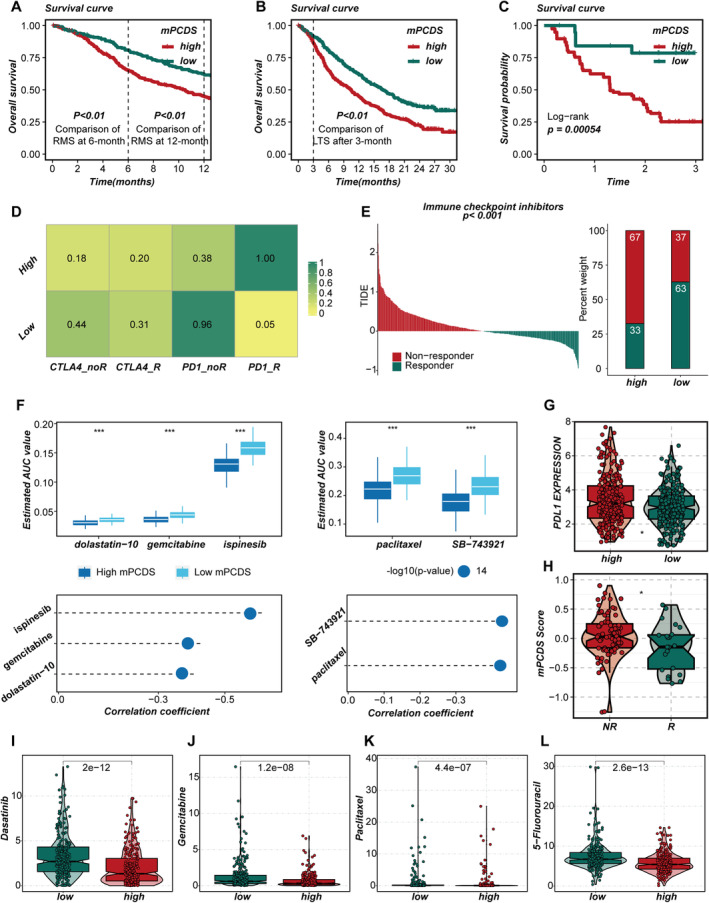
Predictive value of the cell death–related risk signature in immunotherapy response and drug sensitivity. (A) Comparison of restricted mean survival (RMS) at 6 months and 1 year post‐treatment between high and low mPCDS groups within the OAK cohort. (B) Analysis of long‐term survival (LTS) differences at 3 months post‐treatment between high and low mPCDS groups in the OAK cohort. (C) Survival comparison between high and low mPCDS groups in the GSE91061 cohort. (D) Subclass mapping predictions of immunotherapy responses in high and low mPCDS groups. (E) TIDE algorithm predictions of immunotherapy responses in high and low mPCDS groups. (F) Correlation analysis between AUC values of compounds derived from CTRP (right) and PRISM (left) and their distribution across high and low mPCDS groups. (G) Comparative analysis of PDL1 expression levels between high and low mPCDS groups. (H) Distribution of mPCDS scores across different immunotherapy response groups within the GSE91061 cohort. (I–L) Drug sensitivity predictions for high and low mPCDS groups using the ‘oncopredict’ R package (*p < 0.05, **p < 0.01, ***p < 0.001).

To evaluate the role of mPCDS in predicting immunotherapy outcomes, cohorts with available immunotherapy data were examined. In the OAK cohort, the restricted mean survival time at 6 and 12 months was compared between patients in the high‐mPCDS and low‐mPCDS groups, along with long‐term survival differences observed 3 months after treatment. This showed a delayed clinical effect of immunotherapy (*p* < 0.05; Figure 7A,B). It was noted that patients in the low‐mPCDS group had better survival rates, suggesting they benefited more from immunotherapy. Similar results were found in the GSE91061 cohort, where Kaplan–Meier analysis indicated that lower mPCDS scores were linked to improved outcomes after immunotherapy (Figure 7C). To further investigate the different responses to immunotherapy between the high‐ and low‐mPCDS groups, subgroup mapping was performed on a melanoma cohort receiving treatment. Lower mPCDS scores were found to be associated with better responses to PD‐1 therapy (Figure 7D). In addition, the relationship between immune infiltration and immunotherapy response was analysed using the TIDE algorithm and LUAD transcriptome data (Figure 7E). A significant difference in response rates to immunotherapy was observed between the groups. Based on the TIDE results, patients with lower mPCDS scores in LUAD may respond better to immunotherapy. Similar outcomes were seen in the GSE91061 cohort (Figure 7H). These findings highlight that mPCDS scores are helpful not only for predicting prognosis but also for forecasting positive responses to immunotherapy, making mPCDS an important tool for guiding treatment decisions and tailoring therapies for LUAD patients. Interestingly, although higher PD‐L1 expression levels were seen in the high‐mPCDS group (Figure 7G), which is usually linked to better responses to ICIs, our results suggest that PD‐L1 expression alone may not be enough to predict immunotherapy effectiveness. Despite elevated PD‐L1 levels, patients with higher mPCDS scores had poorer prognoses and weaker responses to ICIs.

To explore the connection between mPCDS and drug sensitivity, drug sensitivity and gene expression data from large cancer cell line (CCL) collections in the CTRP and PRISM databases were used to create drug response prediction models. By studying gene expression profiles, the ridge regression model in the ‘pRRophetic’ package was used to calculate AUC values for each compound in clinical samples, with lower AUC values indicating higher drug sensitivity. Drug response differences between high‐ and low‐mPCDS groups were first analysed using a threshold of |log_2_FC| > 0.20 to find compounds with the most significant differences in AUC values. Then, the correlation between AUC values and mPCDS scores was calculated using Spearman correlation, focusing on compounds with correlation coefficients |*R*| > 0.20 in the CTRP and PRISM datasets. Two compounds from the CTRP dataset and three from the PRISM dataset were identified (Figure 7F). A negative correlation between AUC values and mPCDS scores was found for all identified drugs, suggesting they may be more effective for patients in the high‐mPCDS group. Box plots further showed that patients in the high‐mPCDS group had increased sensitivity to drugs like paclitaxel, gemcitabine, dasatinib and 5‐fluorouracil (Figure 7I–L).

### 3.9 Exploring the Role of mPCDS in a Pan‐Cancer Context

**FIGURE 8:**
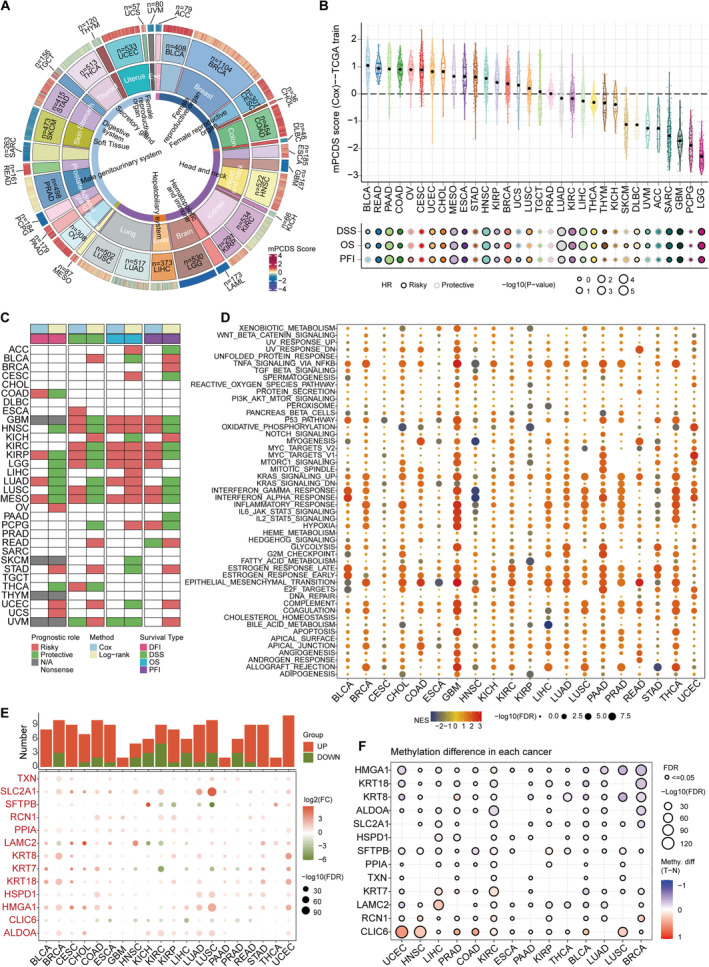
Assessment of the signature's prognostic impact in pan‐cancer analysis. (A) Calculation of mPCDS scores across various tumour types using TCGA pan‐cancer gene expression profiles. (B) Distribution of mPCDS scores across different tumour types and evaluation of the signature's predictive efficacy for overall survival (OS), disease‐specific survival (DSS), disease‐free interval (DFI) and progression‐free interval (PFI). (C) Comprehensive analysis of the correlation between RCN1 expression and patient prognosis, including OS, DSS, DFI and PFI, using univariate Cox regression and Kaplan–Meier analysis. Red indicates that RCN1 is a prognostic risk factor for cancer; green indicates a protective factor. Only results with *p* < 0.05 are reported. (D) Enrichment analysis of Hallmark pathways comparing tumours with high and low mPCDS scores, quantified by normalised enrichment scores (NES) derived from GSEA. (E) The upper panel presents a histogram depicting the count of genes with significant differential expression, while the heatmap illustrates the fold changes and false discovery rates (FDR) of model genes across different cancers. Significantly upregulated genes are shown in red, while downregulated genes are in green. (F) Bubble chart displaying differences in methylation patterns of model genes between tumour and normal tissues across various cancers.

To evaluate the effectiveness of mPCDS across various cancers, the TCGA PanCanAtlas dataset was utilised. mPCDS scores were calculated for samples from 32 different cancers, and the distribution of these risk scores was visualised (Figure 8A,B). Cox regression analysis was conducted to determine whether mPCDS affected OS, DSS and progression‐free survival (PFS) across different cancers. It was found that in 17 of the 32 cancer types, mPCDS had a negative impact on OS, DSS and PFS, suggesting that the mPCDS model could be a broadly applicable prognostic indicator of poor outcomes across multiple cancer types. Interestingly, mPCDS showed a consistent protective effect in cancers such as cervical cancer (CESC), stomach cancer (STAD), testicular germ cell tumours, kidney chromophobe, adrenocortical carcinoma and prostate adenocarcinoma (PRAD). These results suggest that the protective mechanisms at play in these cancers should be investigated further. Gene set enrichment analysis (GSEA) was then applied to examine differences in tumour hallmark gene sets between high and low mPCDS groups (Figure 8D). Pathways such as tumor necrosis factor alpha (TNFA) signalling via nuclear factor kappa B (NFKB), kirsten rat sarcoma viral oncogene homolog (KRAS) signalling and epithelial‐mesenchymal transition were enriched in the high mPCDS group. These pathways provide a molecular foundation for the aggressive characteristics seen in these tumours, which promote cell proliferation, survival and metastasis, all of which contribute to poorer clinical outcomes. Targeting these pathways may improve outcomes for patients with high mPCDS scores. More research is needed to confirm these findings and explore possible therapies for reducing the aggressiveness of high‐risk tumours. The role of model genes in a pan‐cancer context was also examined by analysing their expression across various cancers (Figure 8E). Most of the model genes showed high expression levels in several cancer types, indicating their involvement in tumour development and progression. On the other hand, the expression patterns of genes like SFTPB, LAMC2, KRT7 and CLIC6 varied significantly among different cancer types, suggesting that these genes may play context‐dependent roles. In addition, the methylation levels of model genes were analysed (Figure 8F), showing that most model genes had reduced methylation in tumours, which could lead to their overexpression and contribute to tumour development. Further investigation into the core gene RCN1's prognostic ability in a pan‐cancer context was conducted using Cox modelling and Kaplan–Meier survival analysis (Figure 8C). The heatmap revealed that RCN1 was a risk factor in most cancers. This study underscores the importance of mPCDS in various cancer types and highlights areas for further research into other cancer types.

### 3.10 External Validation of RCN1 Gene Function

**FIGURE 9:**
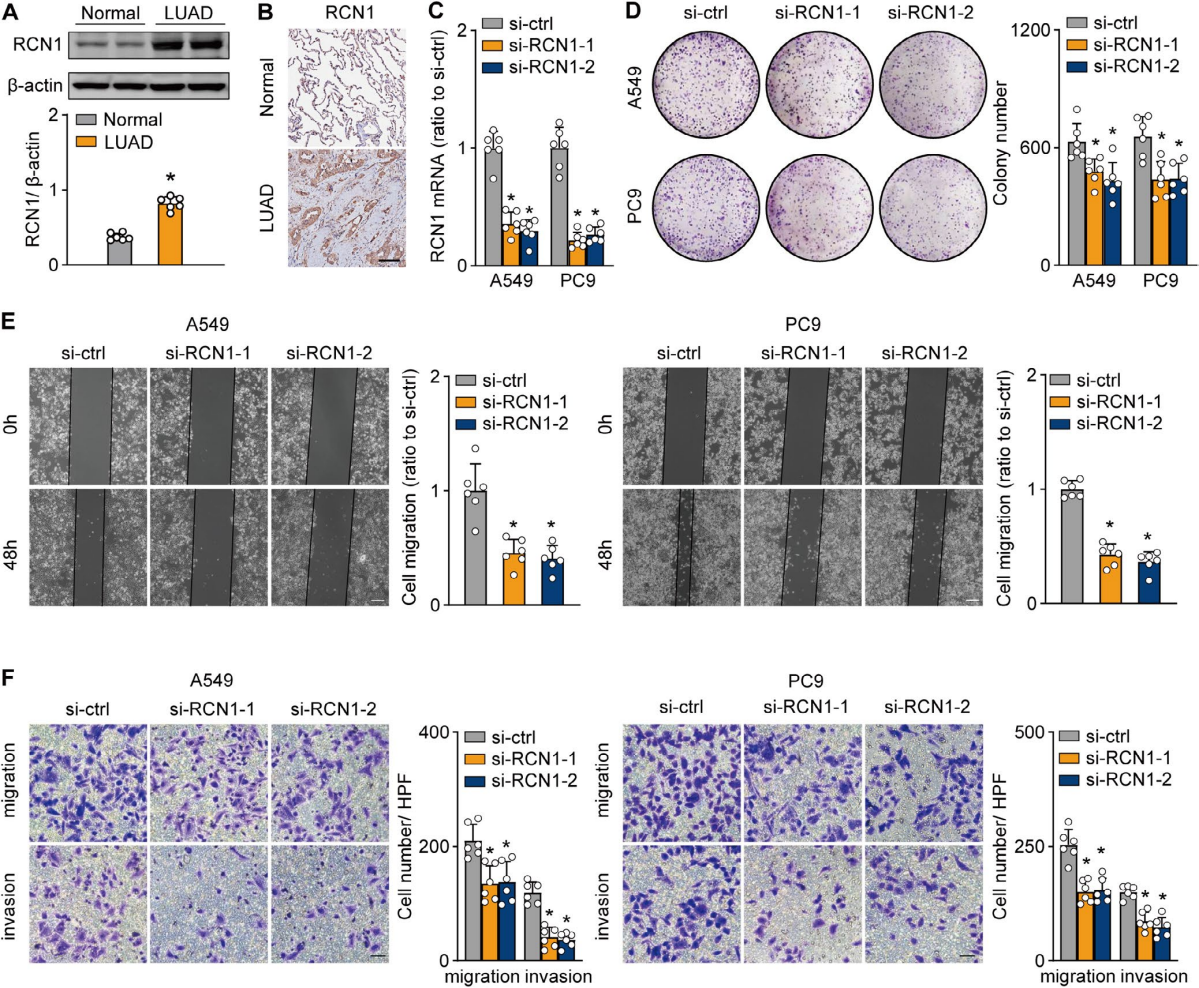
External experimental validation of RCN1 expression and function. (A) Western blot analysis demonstrating RCN1 protein expression levels in normal lung tissue compared to lung cancer tissues. (B) Representative immunohistochemistry images depicting RCN1 staining patterns in both lung cancer and normal tissues. (C) Quantitative RT‐qPCR analysis of RCN1 expression in A549 and PC9 cell lines following transfection with si‐RNA or control, highlighting the relative expression levels. (D) Colony formation assay results showing the impact of RCN1 knockdown on cell proliferation in si‐RNA–transfected cells compared to controls. (E) Scratch‐wound healing assay results, indicating a significant reduction in wound healing rates in cells with downregulated RCN1 expression. (F) Transwell assay results, illustrating that decreased RCN1 expression reduces the migratory and invasive capabilities of A549 and PC9 cells. *P < 0.05.

Our findings showed that elevated RCN1 expression was linked to poor prognosis in LUAD patients, indicating that RCN1 acts as a risk factor in tumours. However, the specific mechanism by which RCN1 drives lung cancer progression is still not fully understood. To examine RCN1 protein levels, Western blot (Figure 9A and Figure S8) and immunohistochemistry (IHC) staining (Figure 9B) analyses were performed on six pairs of LUAD tumour tissues and adjacent normal tissues. RCN1 expression was significantly higher in tumour tissues compared to normal tissues, suggesting that RCN1 may function as an oncogene in lung cancer. To investigate RCN1's role further, siRNA‐mediated knockdown was employed to reduce RCN1 RNA levels in A549 and PC9 cell lines (Figure 9C). The results showed that RCN1 knockdown significantly decreased the proliferation of LUAD cells (Figure 9D). Additionally, the effect of RCN1 inhibition on cell migration and invasion was studied. Quantitative analysis of wound healing assays demonstrated that RCN1 knockdown significantly reduced wound healing rates, indicating that cell migration was significantly impaired (Figure 9E). Moreover, Transwell migration and invasion assays confirmed that the migratory and invasive capabilities of LUAD cells were notably reduced following RCN1 knockdown (Figure 9F). These results emphasise RCN1's critical role in promoting LUAD cell proliferation, migration and invasion, positioning RCN1 as a potential therapeutic target for treating LUAD.

## 4 Discussion

This research is the first to provide a comprehensive analysis of the role played by m6A‐related PCD genes in LUAD risk stratification and clinical management. A prognostic signature consisting of 13 genes (SLC2A1, HSPD1, HMGA1, KRT8, KRT18, ALDOA, RCN1, PPIA, TXN, KRT7, LAMC2, SFTPB and CLIC6) was developed by employing machine‐learning algorithms and integrating extensive RNA sequencing and single‐cell sequencing data. The signature was validated rigorously across multiple independent cohorts, consistently showing strong predictive performance. Significant prognostic value is not only demonstrated by the mPCDS, but its critical role within the TME has also been established. In addition, the exploration of the link between mPCDS and drug sensitivity revealed five possible therapeutic drugs for LUAD patients with elevated mPCDS values. RCN1, identified as a potential oncogene that promotes LUAD progression, was also highlighted as a feasible therapeutic target. These discoveries contribute to the progress of personalised predictive approaches and precision therapies for LUAD, enhancing understanding of this malignancy and improving its clinical management.

With the progression of precision oncology, much focus has been placed on accurately predicting patient survival using various methodologies. Traditional clinical indicators such as tumour size and lymph node metastasis, while still considered independent prognostic factors for LUAD, have shown diminishing predictive ability due to the genetic and genomic heterogeneity observed in LUAD patients [53, 54]. Gene signatures obtained from large‐scale gene expression datasets are considered to have significant potential for assessing survival in cancer patients [55]. Growing recognition has been given to the importance of m6A modification in regulating physiological and pathological processes across several types of cancer, such as pancreatic, breast and colorectal cancers [56]. Though models for PCD have been examined in LUAD prognosis, most focus has been placed on singular mechanisms of cell death, such as apoptosis, autophagy, necrosis, ferroptosis and pyroptosis [57, 58]. However, a clear gap in the current literature remains regarding the connection between m6A modification and PCD in the prognosis of LUAD. This study identified 1054 m6A‐related PCD genes using Spearman correlation analysis, and Cox regression combined with Kaplan–Meier survival analysis identified 146 m6A‐related prognostic PCD genes. This set of genes was used to develop an m6A‐related PCD gene signature. Furthermore, the distribution of m6A‐related PCD genes in single‐cell sequencing datasets was investigated, revealing differences in cellular communication and pathway activation between cells with high and low mPCD activity. Insights into the interactions between m6A modification and PCD at the single‐cell level were provided by this study. By integrating large amounts of RNA sequencing data with single‐cell sequencing data, 43 prognostic genes were identified, which were subsequently used to develop a machine‐learning–based prognostic model.

The use of machine‐learning techniques in predicting patient survival has become increasingly important in advancing personalised medicine. However, considerable challenges are encountered when applying these technologies to clinical practice, particularly regarding model accuracy. Algorithm selection is often influenced by researchers' preferences and biases, leading to a certain level of subjectivity [59]. To address this challenge, our study employed an integrated strategy combining 10 distinct algorithms, ultimately forming 101 different combinations. Following a comprehensive analysis, we identified the combination of CoxBoost and SuperPC as the most effective model, which we named mPCDS. In the early stages of model development, CoxBoost was primarily used for dimensionality reduction and variable selection. However, when applied directly, its predictive accuracy was suboptimal. To overcome these limitations, we integrated the strengths of both CoxBoost and SuperPC. First, CoxBoost was employed to select 13 prognostically relevant genes from the initial gene pool, a crucial step in reducing the risk of overfitting. Subsequently, SuperPC was used to construct the final model, significantly enhancing predictive accuracy. This approach not only reduced model complexity by minimising the number of variables but also improved its generalizability. The common issue of overfitting during model development was addressed by using the average *C*‐index across different cohorts as the primary ranking criterion. In comparison with 106 recently published LUAD prognostic models, our model performed exceptionally well in both training and validation phases. Additionally, mPCDS demonstrated better prognostic accuracy than traditional clinical factors, such as age, sex, T stage, N stage, EGFR status and p53 status. These outcomes highlight the reliability of our model as a prognostic tool for LUAD patients, emphasising its potential utility in clinical decision‐making and its contribution to enhancing the precision of cancer treatment.

Our research into mPCDS and related tumour biological pathways has revealed several key molecular mechanisms that are notably enriched in the high mPCDS LUAD cohort, highlighting its aggressive nature. These mechanisms include enhanced regulation and progression of the cell cycle (E2F targets, G2/M checkpoints, Emi1 phosphorylation), strong DNA replication and initiation processes (pre‐replication complex, regulation of primase activity), maintenance of genomic stability (telomerase protein localization, exogenous points) and efficient cell division and mitosis. These observations are consistent with the functions of the genes incorporated into our model. For instance, HMGA1, a chromatin structural protein, is highly overexpressed in malignant tumours and has been shown to promote tumour growth by increasing cell proliferation and survival, disrupting DNA repair, and causing chromosomal instability. HMGA1 plays a critical role in both tumour initiation and progression, and it also acts as a key regulator of autophagy pathways in cancer cells, contributing to tumour development [60, 61]. In NSCLC, HMGA1 interacts with STMN1 to regulate its phosphorylation levels at Ser16 and Ser38, thereby reducing microtubule stability and promoting tumour metastasis [62]. Similarly, SLC2A1 (also known as GLUT1), which is a key protein involved in cellular energy metabolism, facilitates glucose uptake under low‐glucose conditions, stimulates glycolysis and supports the Warburg effect. Its expression promotes tumour cell proliferation and metastasis, while its high methylation and m6A modification sites influence ferroptosis and autophagy processes, impacting tumour immunity [63, 64, 65]. In vitro experiments demonstrated that the overexpression of SLC2A1 promotes NSCLC cell proliferation, invasion, migration and resistance to paclitaxel [66]. HSPD1 (HSP60), another gene included in our model, functions as a molecular chaperone involved in multiple cellular processes and has been identified as a c‐MYC target gene. This connection indicates a potential role in c‐MYC‐driven transformation and metastasis. Oncogenic effects are exerted through mechanisms involving pathways such as p53, Erk1/2, β‐catenin, DNA damage response and EMT. Additionally, HSPD1 serves as a mediator of intercellular communication, immune regulation and modulation of the TME [67, 68, 69, 70, 71]. Another gene, RCN1, a calcium‐binding protein located in the endoplasmic reticulum (ER) and a member of the CREC family, has been associated with both tumour formation and tumour progression [72]. RCN1 has been shown to be expressed in highly invasive breast CCLs but is absent in less invasive lines. Clinical proteomic studies have identified RCN1 as a possible biomarker for renal cell carcinoma [73]. In hepatocellular carcinoma, RCN1 activates c‐MYC signalling via the IRE1α‐XBP1s pathway [74]. In prostate cancer, RCN1 downregulation induces ER stress, leading to apoptosis in some cells and necroptosis in others, depending on the cell type. This process involves activation of pathways such as PERK, eIF2α and PTEN/AKT, which are key regulators of cell survival [73]. In NSCLC, RCN1 overexpression has been linked to worse prognosis and disease progression, although the regulatory mechanisms driving this association remain unclear [75]. Our findings support these observations, as RCN1 overexpression was found to be associated with lower OS rates in LUAD patients and showed a positive correlation with mPCDS (cor = 0.4, *p* < 0.01). Moreover, our cellular experiments revealed that RCN1 significantly enhances LUAD cell proliferation, migration and invasion. These findings underscore RCN1's crucial role in promoting the invasive behaviour of LUAD cells and suggest that RCN1 may serve as a therapeutic target to counteract high‐risk LUAD progression. These results indicate that, despite potential genomic instability and replication stress, high‐risk LUAD cells have developed sophisticated mechanisms that enable rapid growth, division and survival. Targeting these pathways could provide therapeutic strategies to control and potentially reduce the aggressiveness of high‐risk LUAD.

The TME is recognised as a complex and ever‐changing ecosystem that consists of various cell types, including cancer stem cells and infiltrating immune cells. This intricate system coordinates the production and action of growth factors, inflammatory mediators, cytokines and immune components, which create a supportive environment for tumour development, progression and regulation of treatment responses. Tumour cells often accumulate mutations, acquire adaptive traits and actively alter their surrounding microenvironment to avoid immune surveillance by the host [76]. In our study, the relationship between mPCDS scores and the TME was analysed, uncovering significant differences in immune regulation and cellular infiltration. Higher genetic instability was observed in the high mPCDS group, which was characterised by a greater mutation rate in immune‐regulating genes. This genetic instability implies potential difficulties in responding to immune therapies, as well as lower survival rates due to weakened immune function and the emergence of resistance mechanisms. On the other hand, the markedly higher proportion of CD4+ and CD8+ T cells in the low‐mPCDS group highlights a more active and functional immune microenvironment. CD8+ T cells are central to tumour cell elimination due to their cytotoxic activity, directly targeting and killing cancer cells. Similarly, CD4+ T cells play a crucial supportive role, facilitating the activation and proliferation of CD8+ T cells, as well as coordinating the overall immune response. The elevated levels of both CD4+ and CD8+ T cells in the low‐mPCDS group suggest that these patients benefit from a well‐orchestrated immune surveillance system, which may help in controlling tumour growth more effectively. This higher immune cell infiltration could be indicative of a more favourable tumour immune microenvironment, which is better equipped to recognise and eliminate malignant cells. As a result, the low‐mPCDS group is likely to respond more effectively to immunotherapies, particularly ICB therapies such as PD‐1/PD‐L1 inhibitors, which depend on the reactivation of existing immune responses. These findings emphasise the close relationship between mPCDS expression patterns and immune responses, indicating that mPCDS may play a significant role in LUAD progression through its influence on immune‐related gene networks. This highlights the potential of mPCDS as a crucial factor in shaping LUAD's immune landscape, which has important implications for both prognosis and treatment approaches.

Moreover, our research is the first to propose the clinical use of mPCDS in LUAD, which could aid in developing different treatment strategies. The results suggest that the mPCDS risk model is a reliable predictor of both survival and response to immunotherapy in LUAD patients. Specifically, higher survival rates and better responses to ICIs were observed in patients with low mPCDS scores, a result that was validated in a melanoma cohort. While patients with high mPCDS scores showed elevated PD‐L1 expression, their prognosis remained poor, suggesting that mPCDS offers a more comprehensive evaluation of the effectiveness of immunotherapy. Additionally, mPCDS was linked to drug sensitivity, indicating that drugs such as paclitaxel, gemcitabine, dasatinib and 5‐fluorouracil could be beneficial for patients with high mPCDS scores. This finding has the potential to guide personalised treatment approaches based on mPCDS, allowing therapies to be tailored to individual patient characteristics, thereby improving survival outcomes for LUAD patients.

Therefore, our 13‐gene mPCDS offers significant potential for clinical application, particularly in guiding personalised treatment decisions and enhancing risk stratification in LUAD patients. The model's ability to accurately predict patient outcomes allows for better identification of high‐risk individuals who may benefit from more aggressive therapies, such as targeted treatments or immunotherapies. In contrast, patients with lower mPCDS scores, associated with better prognoses, could avoid overtreatment and the associated side effects, thereby improving their quality of life. Moreover, by incorporating mPCDS into routine clinical assessments, clinicians could stratify patients based on molecular risk profiles, complementing traditional clinical factors like tumour stage and mutation status. Additionally, the model's integration with drug sensitivity analysis highlights its potential in guiding treatment selection. For instance, patients with high mPCDS scores, who are likely to exhibit increased sensitivity to specific chemotherapeutic agents such as paclitaxel and gemcitabine, could be prioritised for these therapies. In immunotherapy, the mPCDS could also predict which patients are more likely to benefit from ICIs, helping to personalise immunotherapy regimens. To facilitate its clinical use, the mPCDS could be incorporated into clinical decision‐support systems, where it can complement standard diagnostic workflows through genomic profiling. This integration would empower clinicians to make more informed, data‐driven decisions, improving patient outcomes by tailoring treatments based on individual risk profiles. However, additional validation in prospective clinical trials will be necessary to further assess the model's effectiveness in real‐world settings and its broader applicability across diverse patient populations. This model not only offers a novel approach to LUAD prognosis but also has the potential to serve as a valuable tool in the era of precision oncology, where treatment is increasingly tailored to the genetic and molecular characteristics of each patient.

While our model demonstrated robust performance in both the training and validation cohorts, several important limitations still need to be addressed. Firstly, the sample size of LUAD cases, along with the variety and depth of follow‐up clinical data, is currently inadequate for a truly reliable internal validation. This limitation underlines the necessity for additional cohort studies to evaluate the broader applicability and accuracy of our prognostic prediction model. Without a more extensive and diverse dataset, the generalizability of the model across different populations and clinical settings remains uncertain. Moreover, the precise molecular mechanisms and biological functions of the 13 m6A‐related PCD genes linked to LUAD have not been fully clarified. The roles these genes play in the pathology of LUAD are complex and not yet completely understood, which calls for deeper experimental investigation. Future research will be required to elucidate how these genes contribute to disease progression, immune evasion and other oncogenic processes in LUAD. By filling these knowledge gaps, the biological underpinnings of our model can be better understood, potentially leading to improved predictive power and new therapeutic targets. To facilitate the clinical application of our model, rigorous validation through multicenter randomised controlled trials will be essential. These trials should incorporate a large number of high‐quality samples, diverse patient populations and comprehensive long‐term follow‐up data to verify the model's effectiveness in real‐world settings. Only through such extensive validation can our model be confidently integrated into clinical practice as a reliable tool for predicting patient outcomes and guiding treatment decisions. In addition, the clinical translation of the model requires the development of user‐friendly tools that enable clinicians to calculate mPCD scores based on routine clinical data, such as RNA sequencing results. Developing such tools will necessitate their integration into existing clinical workflows and training healthcare professionals on how to interpret and apply these scores in clinical decision‐making. Furthermore, while promising potential therapeutic drugs were identified by analysing mPCDS values through the CTRP and PRISM datasets, these findings currently lack experimental backing. The theoretical efficacy of these drugs, although suggested by computational predictions, needs to be tested and confirmed through empirical research. This gap points to the necessity of follow‐up studies that focus on experimental validation, ensuring that these identified compounds truly have clinical relevance and can be used to enhance the personalised treatment of LUAD patients.

## 5 Conclusion

In summary, we developed a 13‐gene risk signature based on m6A‐related PCD genes, which demonstrates strong predictive capability for survival outcomes and drug sensitivity in LUAD patients. Notably, RCN1 was identified as a critical oncogene that significantly influences cancer progression and serves as a promising therapeutic target. While these findings warrant further validation in diverse LUAD cohorts, they have the potential to provide novel prognostic biomarkers and insights for personalised prediction methods and precision treatment in LUAD, ultimately guiding individualised therapeutic strategies to improve patient outcomes.

## Author Contributions

**Xiao Zhang:** formal analysis (equal), methodology (equal), validation (equal), writing – original draft (equal). **Yaolin Cao:** software (equal), writing – review and editing (equal). **Jiatao Liu:** data curation (equal), resources (equal). **Qiuyue Yan:** supervision (equal), visualization (equal). **Wei Wang:** conceptualization (equal). **Zhibo Wang:** conceptualization (equal), investigation (equal), project administration (equal), supervision (equal).

## Ethics Statement

Ethics approval was obtained from the Ethics Committee of the First Affiliated Hospital of Nanjing Medical University (no. 2023‐SR‐777). Written informed consent was obtained from all participants regarding the collection of samples, the purpose of the study and the possibility of publication. This was done in line with the ethical guidelines of the First Affiliated Hospital of Nanjing Medical University.

## Conflicts of Interest

The authors declare no conflicts of interest.

## Supporting information


**Figure S1.** Expression characteristics of m6A regulatory factors in lung adenocarcinoma. (A) Box plot illustrating the expression levels of m6A regulatory factors in LUAD tissues compared to normal control tissues within the TCGA‐LUAD dataset. (B) Univariate Cox regression and Kaplan–Meier survival analysis of m6A regulatory factors, assessing their impact on overall survival (OS), disease‐specific survival (DSS), disease‐free interval (DFI) and progression‐free interval (PFI). (C) Correlation analysis of m6A regulatory factors, with significance levels indicated as *p < 0.05, **p < 0.01, ***p < 0.001.
**Figure S2.** Identification of cell types in single‐cell RNA‐Seq data of LUAD. (A) tSNE plot categorising single‐cell RNA‐seq data into 14 distinct subclusters. (B) Bubble plot displaying the expression of marker genes across different cell types. (C) tSNE plot classifying single‐cell RNA‐seq data into 10 distinct cell types. (D) Stacked bar chart illustrating the proportion of different cell types across various samples.
**Figure S3.** Cell interaction analysis between high and low mPCD activity groups. (A) Analysis using CellChat demonstrates an increased frequency and intensity of cell communications within the high mPCD activity group. (B) A network diagram illustrating the patterns of cell‐to‐cell communication within both high and low mPCD activity groups. (C) Comparative analysis highlights the differences in cell communication patterns between the high and low mPCD activity groups. (D) Examination of signalling pathway patterns reveals distinct differences between the high and low mPCD activity groups.
**Figure S4.** (A–G) Survival differences between high and low mPCDS groups in TCGA cohort, GSE11969, GSE13213 cohort, GSE26939 cohort, GSE29016 cohort, GSE31210 and GSE72094. (H–N) PCA analysis of the model in TCGA cohort, GSE11969, GSE13213 cohort, GSE26939 cohort, GSE29016 cohort, GSE31210 and GSE72094.
**Figure S5.** ROC curves of the model in TCGA cohort, GSE11969, GSE13213 cohort, GSE26939 cohort, GSE29016 cohort, GSE31210 and GSE72094.
**Figure S6.** Correlation between model gene expression and mPCDS score.
**Figure S7.** Survival analysis of model genes in the TCGA cohort.
**Figure S8.** Unedited western blot images.

**Table S1.** List of 18 patterns of PCD and key regulatory genes.

**Table S2.** Prior signatures included in this study.

**Table S3.** List of siRNAs used to knock out the indicated target.

**Table S4.** *C*‐index of PCDS and published signatures across various datasets.

## Data Availability

The datasets used and analysed in this research are publicly available through the TCGA repository (http://cancergenome.nih.gov/) and the GEO database (https://www.ncbi.nlm.nih.gov/geo/).
