# Supplementary material for: Comprehensive Analysis of m6A‐Related Programmed Cell Death Genes Unveils a Novel Prognostic Model for Lung Adenocarcinoma
Source: J Cell Mol Med. 2025 Jan 19;29(2):e70255. doi: 10.1111/jcmm.70255 (PMC11743404; doi:10.1111/jcmm.70255)

**Supplemental information**

**Comprehensive Analysis of m6A-Related Programmed Cell Death Genes Unveils a Novel Prognostic Model for Lung Adenocarcinoma**

**Xiao Zhang^1†^, Yaolin Cao^1†^, Jiatao Liu^1†^,** **Wei Wang^1^, Qiuyue Yan^2*^, Zhibo Wang^1*^**

^1^Department of Thoracic Surgery, The First Affiliated Hospital of Nanjing Medical University, Nanjing, 210029, China

^2^Department of Respiratory Diseases, The Affiliated Huai’an Hospital of Xuzhou Medical University, Huai’an, Jiangsu 223002, China.

**^†^**These authors have contributed equally to this work and share first authorship

*** Correspondence:**

Qiuyue Yan, 351124959@qq.com;

Zhibo Wang, wangzhibo0107@yeah.net

**Figure S1. Expression characteristics of m6A regulatory factors in lung adenocarcinoma**

**(A)** Box plot showing the expression levels of m6A regulatory factors in LUAD and normal control tissues in the TCGA-LUAD dataset. **(B)** Univariate Cox regression and Kaplan-Meier survival analysis of m6A regulatory factors for overall survival (OS), disease-specific survival (DSS), disease-free interval (DFI), and progression-free interval (PFI). **(C)** Correlation analysis of m6A regulatory factors. (*p < 0.05, **p < 0.01, ***p < 0.0
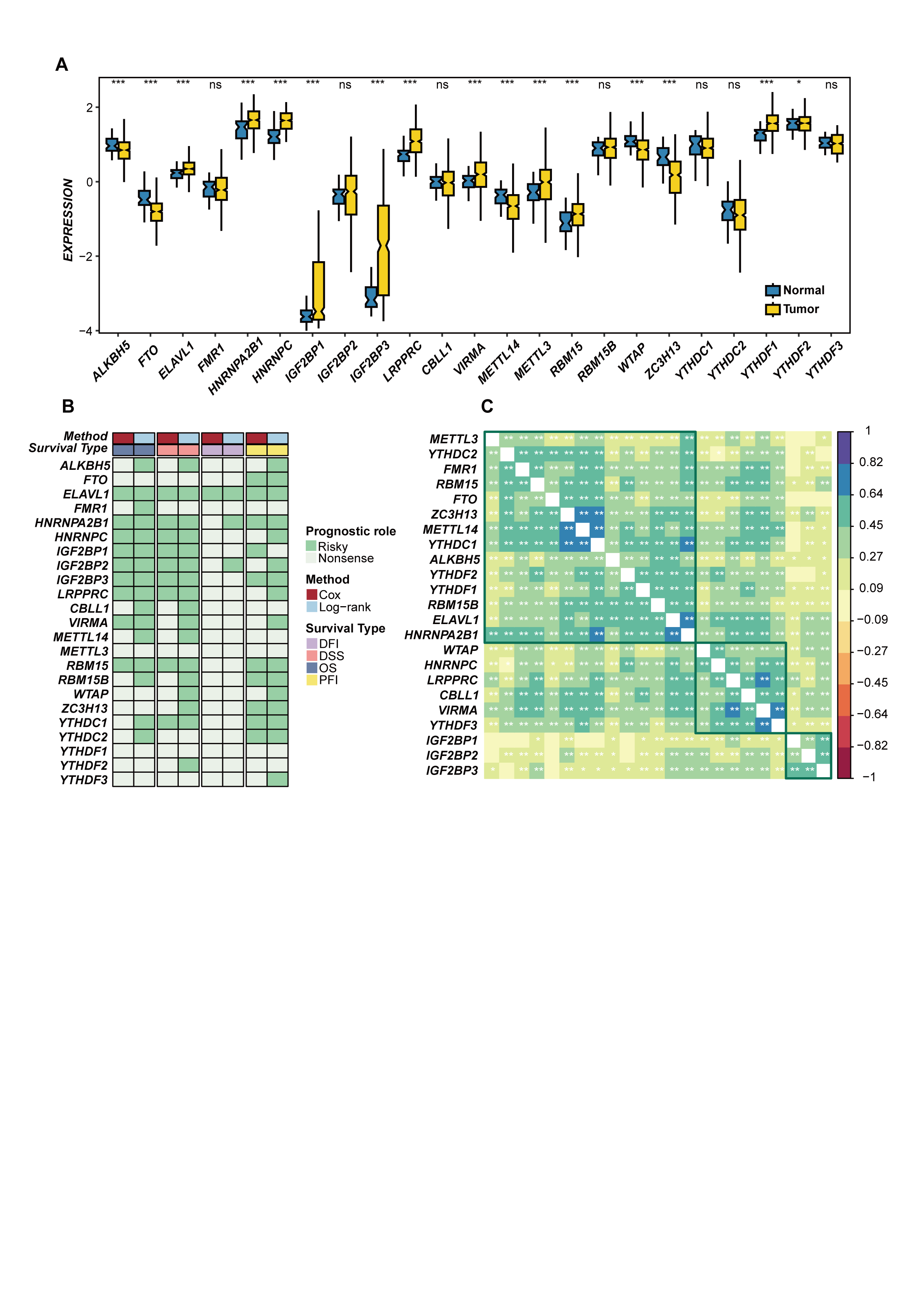


**Figure S2. Identification of cell types in single-cell RNA-seq data of LUAD**

**(A)** tSNE plot segregating single-cell RNA-seq data into 14 subclusters. **(B)** Bubble plot showing the expression of marker genes in different cell types. **(C)** tSNE plot segregating single-cell RNA-seq data into 10 distinct cell types. **(D)** Stacked bar chart representing the proportion of different cell types in various samples.


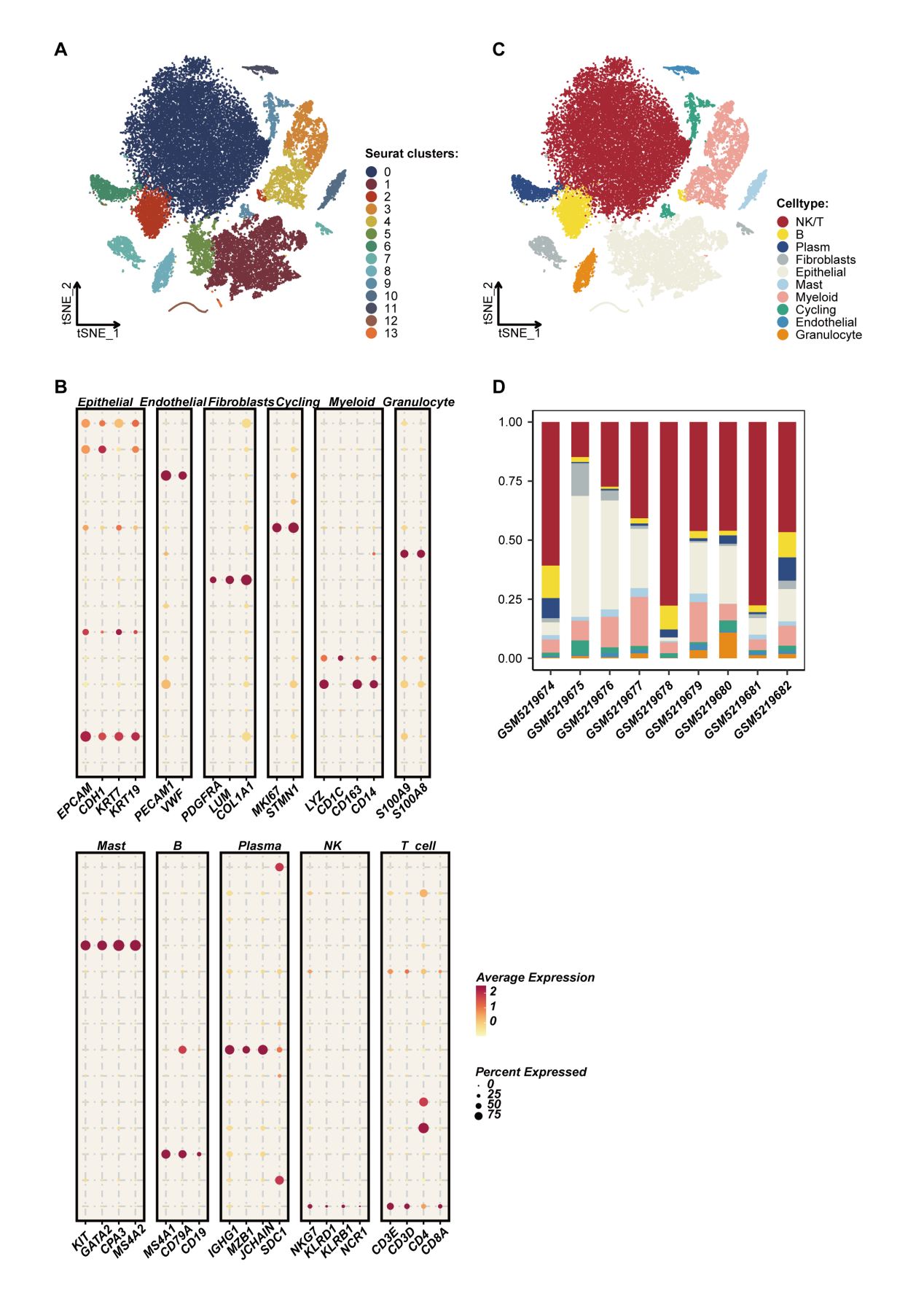


**Figure S3. Cell interaction analysis between high and low mPCD activity groups**

**(A)** CellChat analysis showing increased number and intensity of cell communications within the high mPCD group. **(B)** Network diagram of cell-to-cell communication in high and low mPCD activity groups. **(C)** Differences in cell communication patterns between high and low mPCD activity groups. **(D)** Differences in signaling pathway patterns between high and low mPCD activity groups.


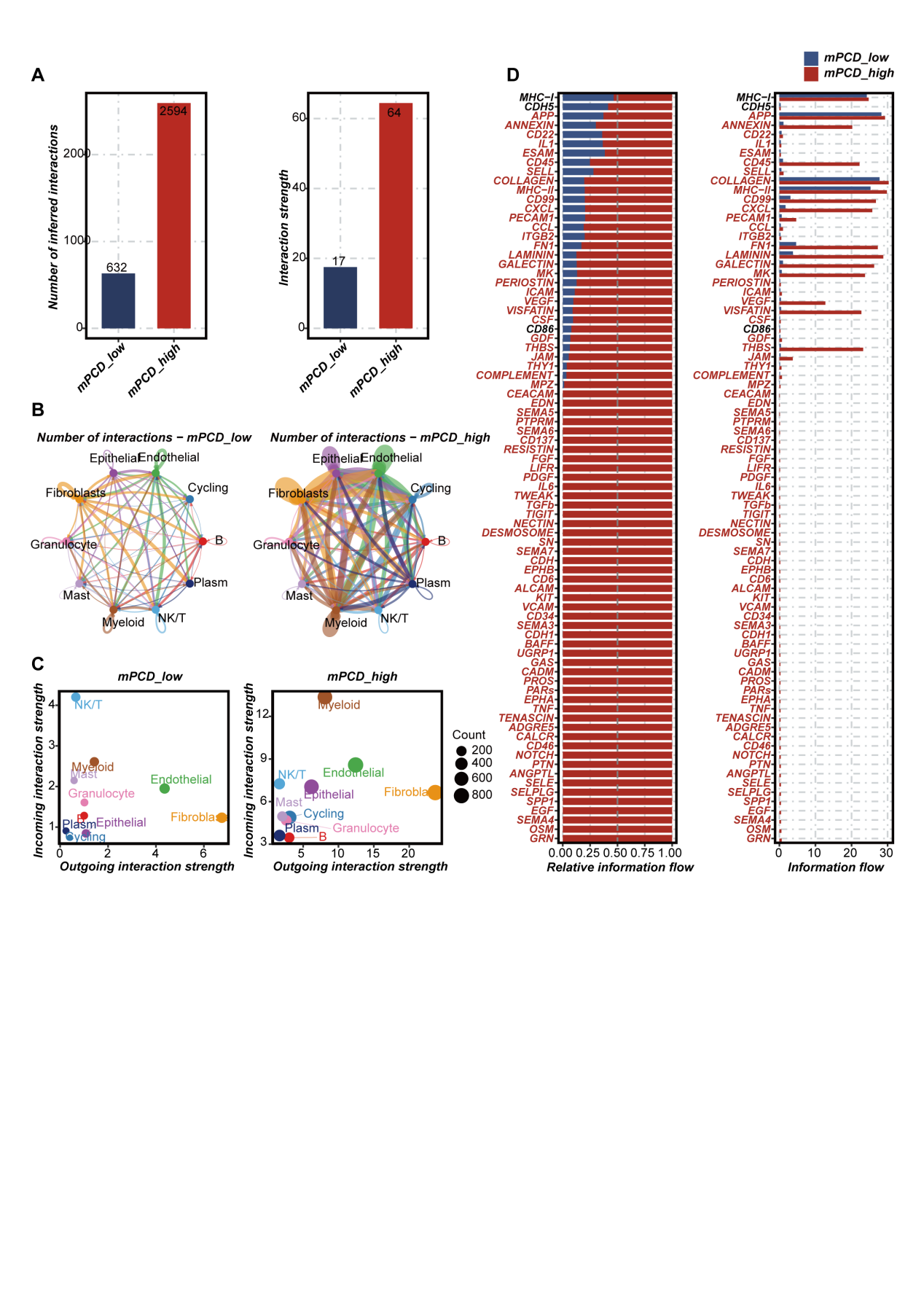


**Figure S4A-G. Survival differences between high and low mPCDS groups in TCGA cohort, GSE11969, GSE13213 cohort, GSE26939 cohort, GSE29016 cohort, GSE31210, and GSE72094.**

**Figure S4H-N. PCA analysis of the model in TCGA cohort, GSE11969, GSE13213 cohort, GSE26939 cohort, GSE29016 cohort, GSE31210, and GSE72094.**


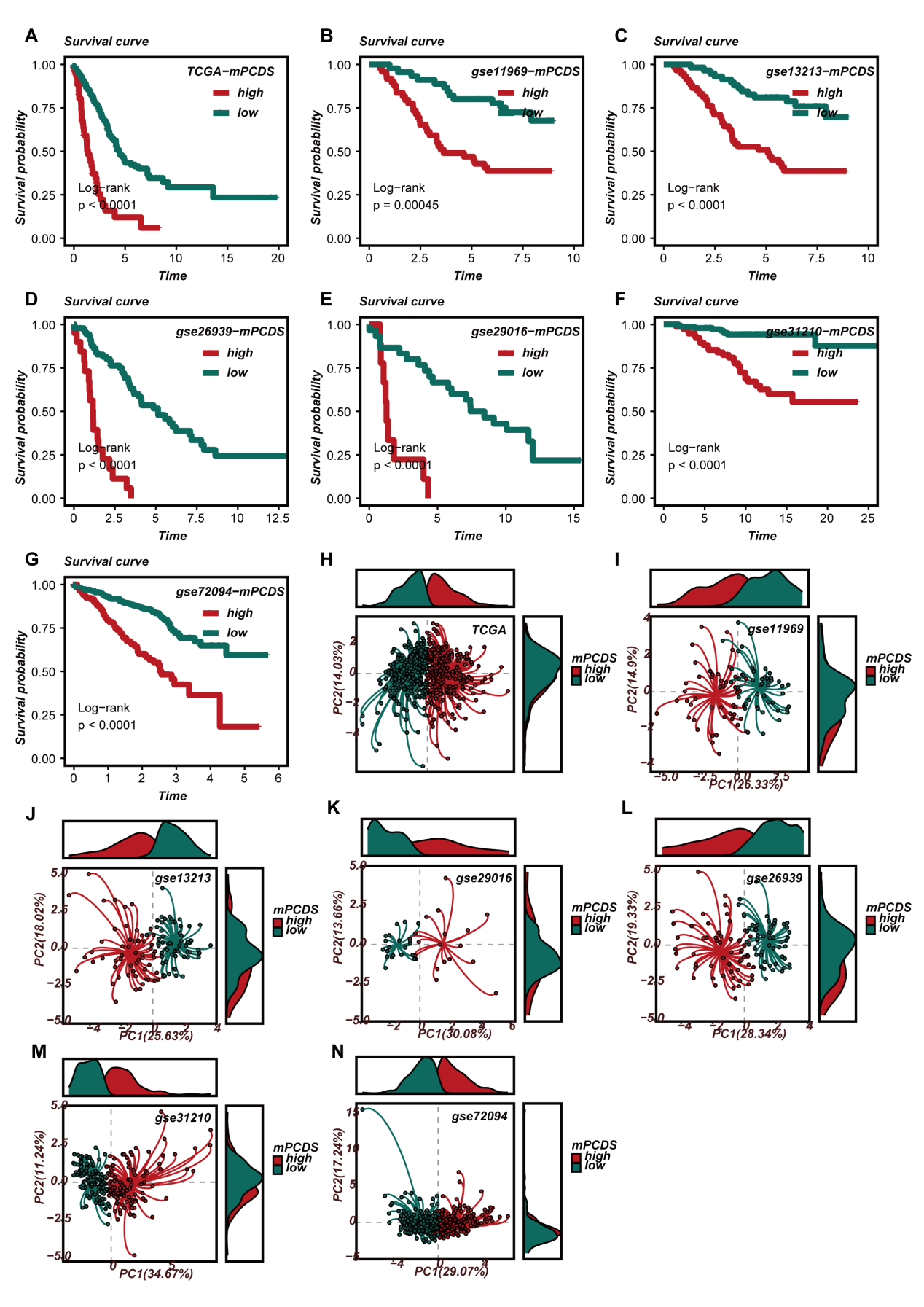


**Figure S5. ROC curves of the model in TCGA cohort, GSE11969, GSE13213 cohort, GSE26939 cohort, GSE29016 cohort, GSE31210, and GSE72094.**


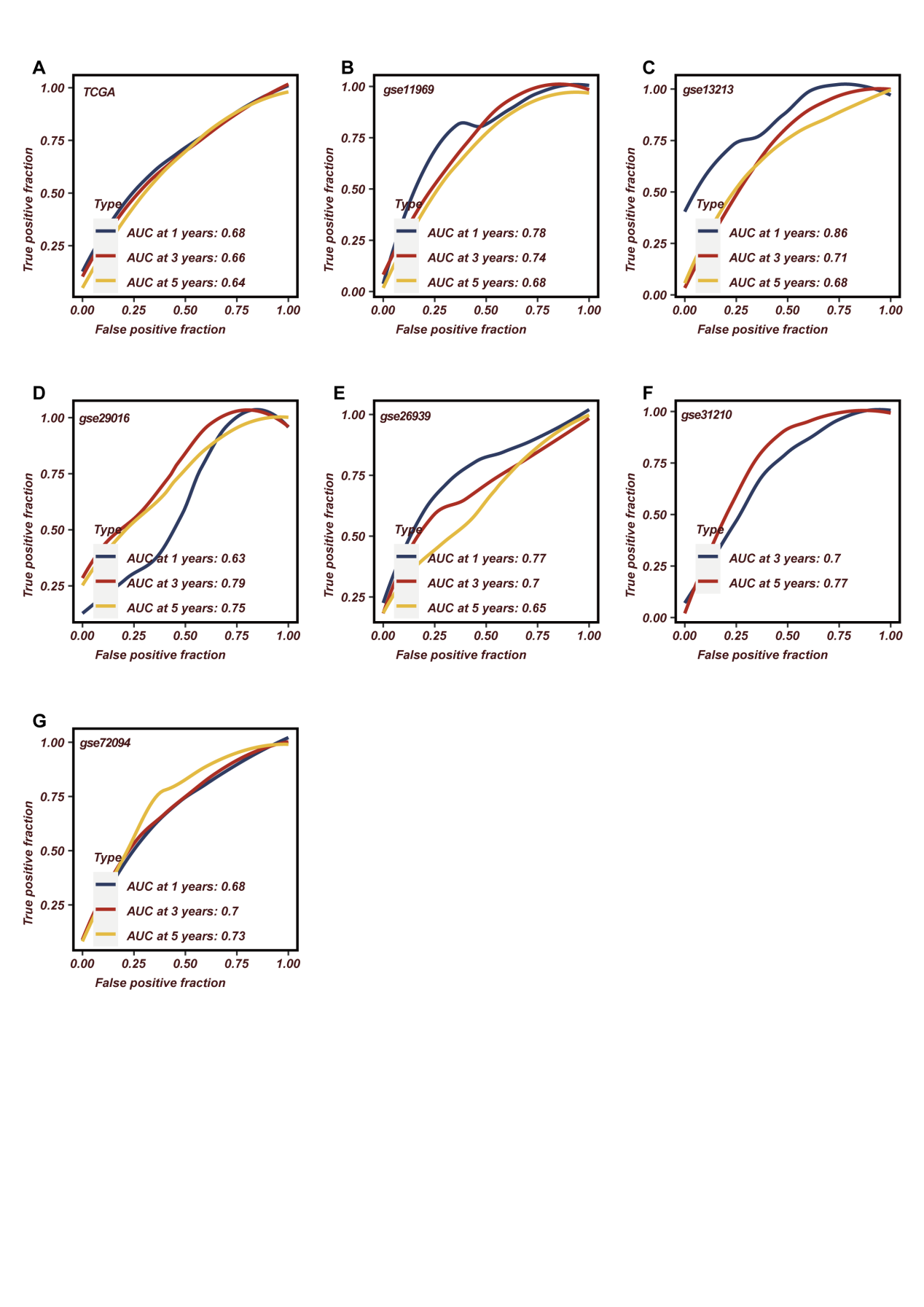


**Figure S6. Correlation between model gene expression and mPCDS score.**


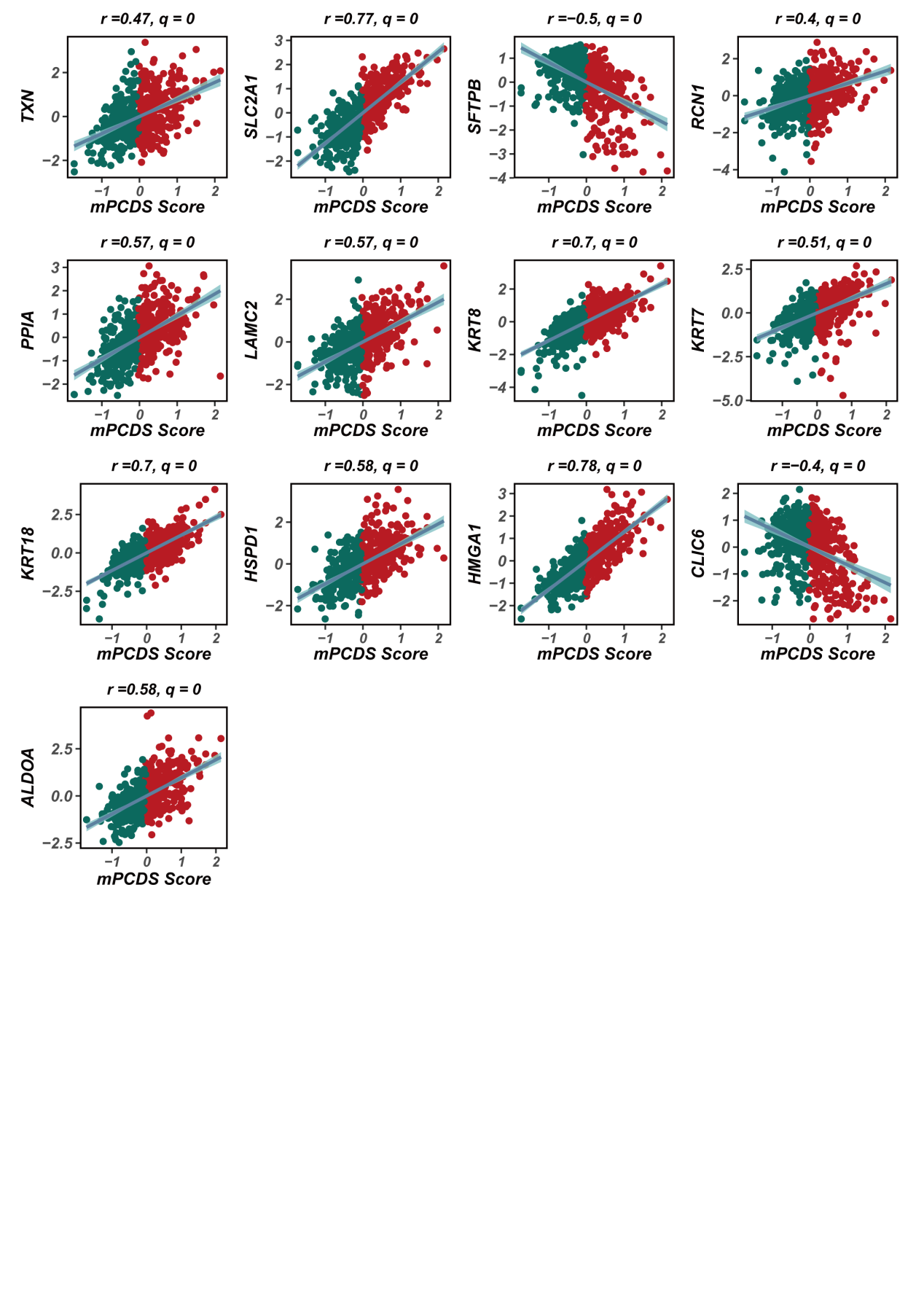


**Figure S7. Survival analysis of model genes in the TCGA cohort.**


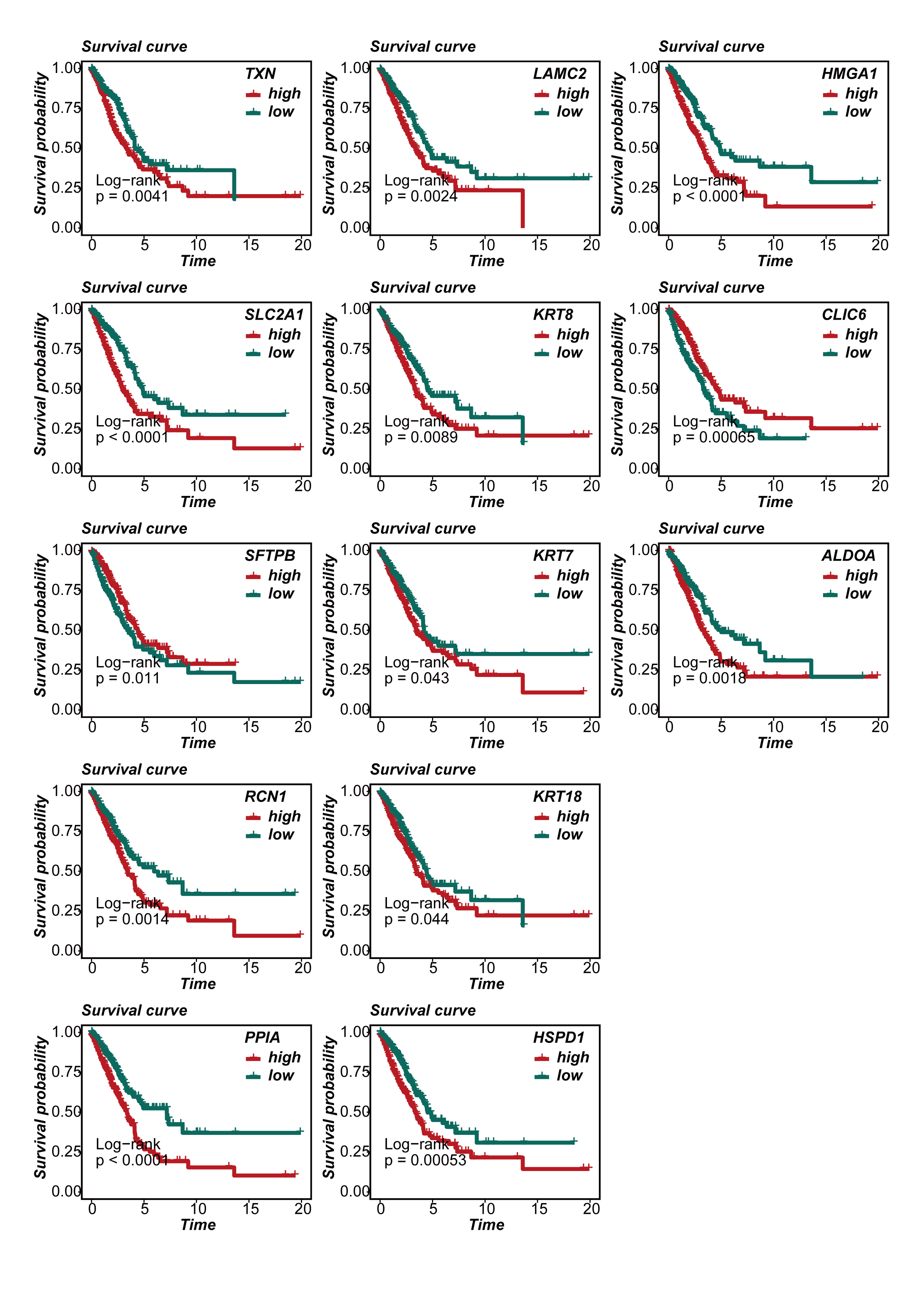


**Figure S8. Unedited western blot images.**


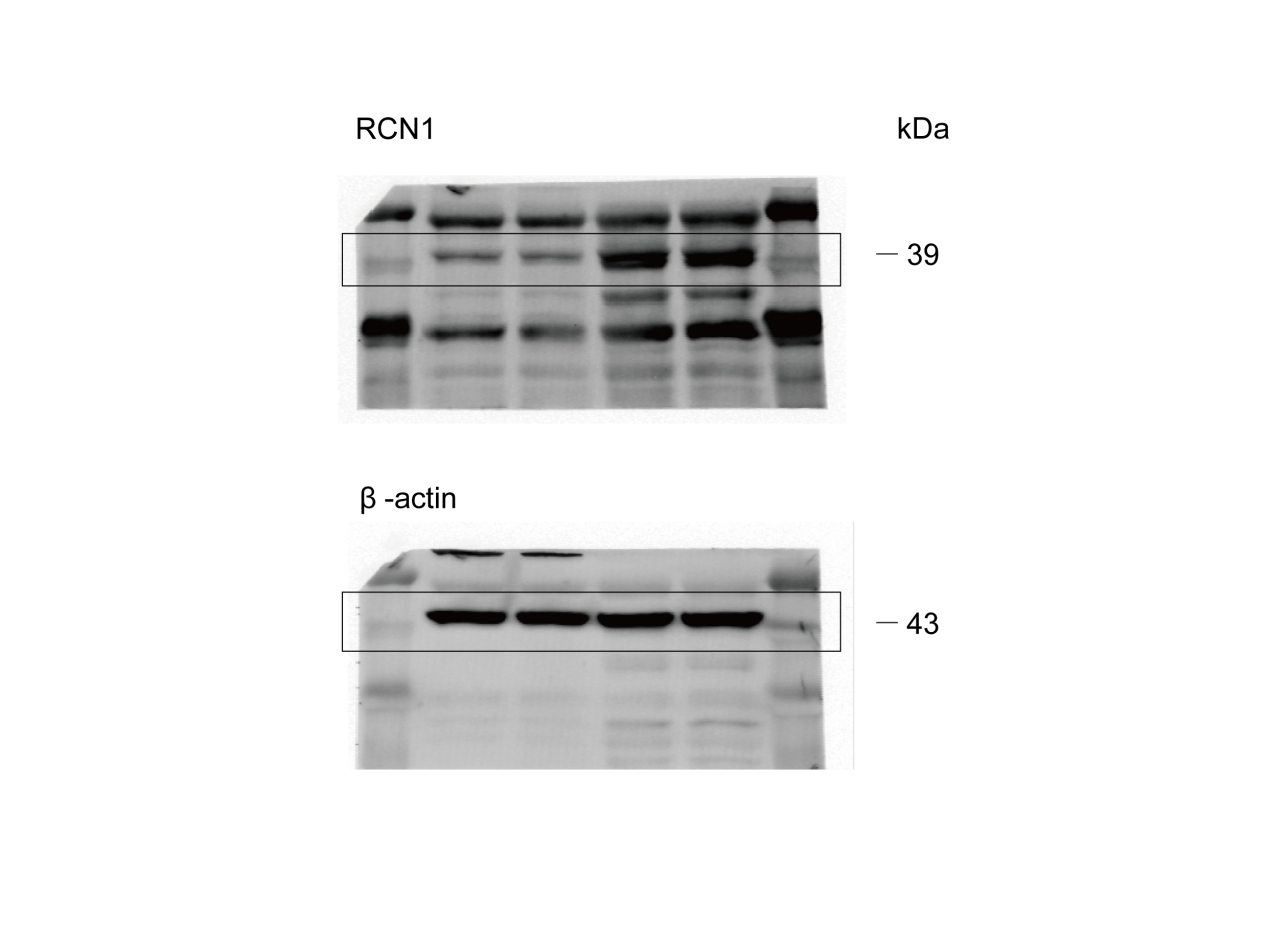

Supplement: Supplementary file 1 — Figure S1. Expression characteristics of m6A regulatory factors in lung adenocarcinoma. (A) Box plot illustrating the expression levels of m6A regulatory factors in LUAD tissues compared to normal control tissues within the TCGA‐LUAD dataset. (B) Univariate Cox regression and Kaplan–Meier survival analysis of m6A regulatory factors, assessing their impact on overall survival (OS), disease‐specific survival (DSS), disease‐free interval (DFI) and progression‐free interval (PFI). (C) Correlation analysis of m6A regulatory factors, with significance levels indicated as *p < 0.05, **p < 0.01, ***p < 0.001. Figure S2. Identification of cell types in single‐cell RNA‐Seq data of LUAD. (A) tSNE plot categorising single‐cell RNA‐seq data into 14 distinct subclusters. (B) Bubble plot displaying the expression of marker genes across different cell types. (C) tSNE plot classifying single‐cell RNA‐seq data into 10 distinct cell types. (D) Stacked bar chart illustrating the proportion of different cell types across various samples. Figure S3. Cell interaction analysis between high and low mPCD activity groups. (A) Analysis using CellChat demonstrates an increased frequency and intensity of cell communications within the high mPCD activity group. (B) A network diagram illustrating the patterns of cell‐to‐cell communication within both high and low mPCD activity groups. (C) Comparative analysis highlights the differences in cell communication patterns between the high and low mPCD activity groups. (D) Examination of signalling pathway patterns reveals distinct differences between the high and low mPCD activity groups. Figure S4. (A–G) Survival differences between high and low mPCDS groups in TCGA cohort, GSE11969, GSE13213 cohort, GSE26939 cohort, GSE29016 cohort, GSE31210 and GSE72094. (H–N) PCA analysis of the model in TCGA cohort, GSE11969, GSE13213 cohort, GSE26939 cohort, GSE29016 cohort, GSE31210 and GSE72094. Figure S5. ROC curves of the model in TCGA cohort, GSE11969, GSE132 [file JCMM-29-e70255-s005.docx]
